# Humanized monoacylglycerol acyltransferase 2 mice on a high-fat diet exhibit impaired liver detoxification during metabolic dysfunction-associated steatotic liver disease

**DOI:** 10.1371/journal.pone.0334213

**Published:** 2025-10-15

**Authors:** J. Jose Corbalan, Pranavi Jagadeesan, Joseph T. Nickels

**Affiliations:** 1 The Institute of Metabolic Disorders, Genesis Research and Development Institute, Hamilton, New Jersey, United State of America; 2 Rutgers Center for Lipid Research, New Jersey Institute for Food, Nutrition, and Health, Rutgers University, New Brunswick, New Jersey, United State of America; St Jude Children's Research Hospital, UNITED STATES OF AMERICA

## Abstract

Obesity significantly increases the risk of hyperlipidemia, type 2 diabetes, and liver disease. This study examined humanized monoacylglycerol acyltransferase 2 mice (*HuMgat2*) and their response to a high fat diet (HFD) while investigating hepatocyte dysfunction during obesity development. *HuMgat2* mice fed a HFD exhibited hyperlipidemia, hyperglycemia, insulin resistance, and metabolic dysfunction-associated steatotic liver disease (MASLD). Elevated levels of cholesterol and triglycerides were associated with increased expression of lipogenic genes and accumulation of nuclear Srebp1/Srebp2. Mice fed a HFD demonstrated impaired insulin signaling and increased glucose production through the expression of gluconeogenesis genes. Liver fibrosis was characterized by collagen deposition and activation of Jak2-Stat3 signaling, resulting in hepatocyte apoptosis. RNA sequencing identified extracellular matrix degradation and apolipoprotein metabolism as being altered. Levels of cytochrome P450 enzymes were downregulated, as indicated by decreased Cyp2b10 and Cyb3a11 levels, alongside reduced expression of the di- and tri-carboxylic acid transporter Slc13a2, correlating with elevated Krebs cycle intermediates. Notably, *HuMgat2* mice exhibited responses to a high-fat diet that were comparable to those observed in *mMgat2* mice. These findings suggest that HFD consumption and concomitant obesity disrupts metabolite homeostasis, contributing to liver damage and cell death. They also further validate *HuMgat2* mice as an excellent preclinical model for testing human MOGAT2 inhibitors as therapeutics for treating obesity.

## Introduction

Between 30–50% of the world’s population is obese [1]. Obesity is identified as a risk factor for metabolic diseases such as hyperlipidemia, type 2 diabetes (T2D), cardiovascular disease (CVD), and metabolic dysfunction-associated steatotic liver disease (MASLD) [2]. Health care costs related to obesity are estimated at $150 billion dollars annually [3]. An important issue is the increasing rate of obesity in children [4].

Obesity has significant effects on cell metabolite biochemistry [5,6]. Metabolomics analyses using blood from individuals with obesity have linked high BMI with alterations in the TCA cycle, tryptophan pathway, and urea cycle [5,7]. Blood from individuals with insulin resistance, dyslipidemia, hyperglycemia, and high adiposity show similar metabolic profiles [8]. Individuals with obesity exhibit elevated levels of branch-chained amino acids [9]. TCA cycle intermediates such as succinate and citrate are also increased [8,10,11], and oxaloacetate in adipose tissue can contribute to fatty acid synthesis [12]. Mice lacking the Slc13a5 citrate transporter display resistance to diet-induced obesity, and inhibitors have been developed to target several Slc13a family members including Slc13a5 [13]. During obesity, anions like sulfate can accumulate, leading to the expression of sulfotransferases, sulfatases, and PAPS synthases that regulate key metabolic pathways.

Various dietary regimens can profoundly influence overall metabolism, affecting numerous facets of health such as weight management, metabolic health indicators, and susceptibility to diseases [14–16]. Esko *et al.* [17] observed significant variations in the levels of numerous metabolite intermediates, including Krebs cycle substrates, lipids, and amino acids, depending on whether individuals with obesity followed a low-fat, low glycemic index, or very-low carbohydrate diet. Current research has begun to focus on mapping diet-related abnormal metabolic signatures with specific disease states like CVD and specific cancers [18–20]. Rafiq et al. [21]conducted a study analyzing metabolites from specific foods, connecting long-term consumption with specific diseases and identifying nutritional biomarkers.

Cells have developed detoxification pathways activated under various conditions to address metabolite toxicity [22–24]. Toxic drugs, xenobiotics, and lipids activate cytochrome P450 enzymes [25]. *Cyp* gene expression is induced by the activation of the constitutive androstane receptor, whose ligands include bile acids, oxysterols, androgens, and certain medications [23]. Nuclear pregnane X receptor signaling is also activated, inducing bile acid (*Cyp7a1*, *Cyp8a1*, and *Cyp3a4*), multi-drug resistance pump (*Mdr1*), and sulfotransferase (*Sult1e1*) gene expression [22]. Obesity can lead to the circumvention or overwhelming of these pathways [26–29].

Human MOGAT and mouse Mgat2 enzymes catalyze the conversion of monoacylglycerols and fatty acyl-CoAs to diacylglycerides, which are subsequently converted to triacylglycerides by diacylglycerol acyltransferases [30]. Humans possess three MOGATs (MOGAT1, MOGAT2, MOGAT3) that belong to the DGAT2 family [31]. Human MOGAT2 shares 81% homology with mMGAT2. Microsomes containing human MOGAT2 or mouse Mgat2 enzymes have demonstrated equivalent affinities for adding fatty acids to the *sn2* position of monoacylglycerol with comparable kinetics and substrate specificities. Human MOGAT2 activity is present in the liver and the duodenum of the small intestine, where it plays a crucial role in the resynthesis of dietary triglycerides that are transported by chylomicrons to the liver [32].

*Mgat2*^*-/-*^ mice on a high-fat diet are resistant to obesity and fatty liver, show improved glucose tolerance, and have higher energy expenditure [33]. Expressing human *MOGAT2* in the intestines of knockout animals, restores phenotypes, indicating that the intestinal MOGAT2 enzyme is crucial for whole body triglyceride homeostasis in mice, and thus humans [34]. Interestingly, ablating *mMgat2* expression in adult mice fed a high fat diet reverses obesity, suggesting that human MOGAT2 may represent a viable target for treating the disease [35].

We developed a humanized *HuMgat2* mouse model expressing *hMOGAT2* to optimize our human MOGAT2 inhibitors. Our recent studies have shown that *HuMgat2* mice develop MASLD/MASH when fed a steatotic diet, demonstrating metabolic phenotypes like those observed in wild type m*Mgat2 m*ice [36]. These results indicate that the *HuMgat2* model can serve as an effective pre-clinical drug discovery tool and provide insights into the mechanisms by which obesity contributes to MASLD/MASH.

In the current study, we subjected *HuMgat2* mice to a high-fat diet (HFD) to investigate how obesity triggers the transition from MASLD to MASH, while also validating the model for lead optimization of our human MOGAT2 inhibitors as anti-obesity therapeutics. Our findings demonstrate that *HuMgat2* mice on a high-fat diet exhibit obesity, glucose intolerance, insulin resistance, and early indications of MASH. Furthermore, our results suggest that impaired liver detoxification plays a significant role in the development of these high-fat diet- and obesity-related phenotypes.

## Results

### *mMgat2* and *HuMgat2* mice exhibit obesity when fed a high-fat diet (HFD)

**Fig 1:**
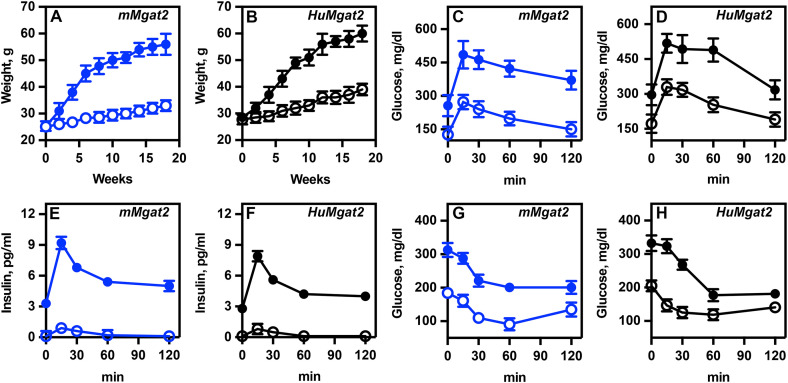
*mMgat2* and *HuMgat2* mice become obese when fed a HFD. A & B, *mMgat2* (blue circles) and B, *HuMgat2* (black circles) mice were fed a chow (open circles) diet or HFD (filled circles) for 16 weeks, with body weights measured weekly (n=8). C & D, an OGTT was performed at 12 weeks. Mice were fasted for 16 hours prior to study initiation. Glucose levels were measured at specified times following a 2g/kg body weight glucose bolus (100mg/ml) given by oral gavage. E & F, insulin levels were measured at specific time points during the oral glucose tolerance test. G & H, glucose levels were measured at specific times after an intraperitoneal bolus of 0.5 units/kg and 1.0 units/kg of insulin for chow-fed and HFD-fed mice, respectively. For body weight measurements, a two-way ANOVA analysis with Dunnett's post hoc test was used to compare chow diet-fed mice. For all other experiments, a two-way ANOVA analysis with Tukey's post hoc test was used. Values are mean ± S.D.

*HuMgat2* and *mMgat2* mice were given either a standard chow diet or a high-fat diet for 16 weeks. Their body weights were recorded weekly. Minimal weight gain occurred in chow-fed *mMgat2* (Fig 1A, open blue circles) and *HuMgat2* (Fig 1B, open black circles) mice. However, mice fed a high-fat diet gained weight over time, doubling their body weight by the end of the study (Fig 1A, B; *mMgat2*, closed blue circles; *HuMgat2*, closed black circles).

### *mMgat2* and *HuMgat2* mice exhibit glucose intolerance and insulin resistance when fed a HFD

We subsequently investigated whether obesity induced glucose intolerance and insulin resistance. An Oral Glucose Tolerance Test (OGTT) was performed at 12 weeks, followed by an Insulin Tolerance Test (ITT) at 14 weeks.

*mMgat2* (Fig 1C, open blue circles) and *HuMgat2* (Fig 1D, open black circles) mice that were fed a chow diet showed an increase in glucose levels at 15 minutes, which gradually returned to baseline by 120 minutes, indicating a normal glucose excursion rate. Both cohorts on the HFD had higher fasting glucose levels than those on a chow diet (Fig 1C, D; *mMgat2*, closed blue circles; *HuMgat2*, closed black circles). After glucose administration, glucose levels increased significantly to around 500 mg/dl at 15 minutes (Fig 1C, D; *mMgat2*, closed blue circles; *HuMgat2*, closed black circles). Elevated levels persisted for 60 minutes before gradually returning to fasting levels by 120 minutes.

*mMgat2* and *HuMgat2* mice on a chow diet exhibited low levels of insulin secretion during the OGTT (Fig 1E, F; *mMgat2*, open blue circles; *HuMgat2*, open black circles). Insulin secretion was significantly higher in both groups when fed the HFD (Fig 1E, F; *mMgat2*, closed blue circles; *HuMgat2*, closed black circles). *mMgat2* and *HuMgat2* mice fed the HFD exhibited insulin resistance, demonstrated by higher sustained glucose levels during glucose infusion in the ITT (Fig 1G, H; *mMgat2*, closed blue circles; *HuMgat2*, closed black circles).

### Obese *mMgat2* and *HuMgat2* mice have defects in liver insulin signaling

Based on our OGTT and ITT results, we hypothesized that insulin signaling might be impaired in obese *HuMgat2* mice. Insulin receptor signaling induces AKT phosphorylation at Ser^473^ by mTORC2 and ribosomal protein S6 phosphorylation at Ser^235/236^ by p70S6 kinase [37]. We measured the phosphorylation levels of Akt and S6, as well as the downstream insulin signaling targets, Gsk3β, and 4E-BP1 using ELISA assays.

**Fig 2:**
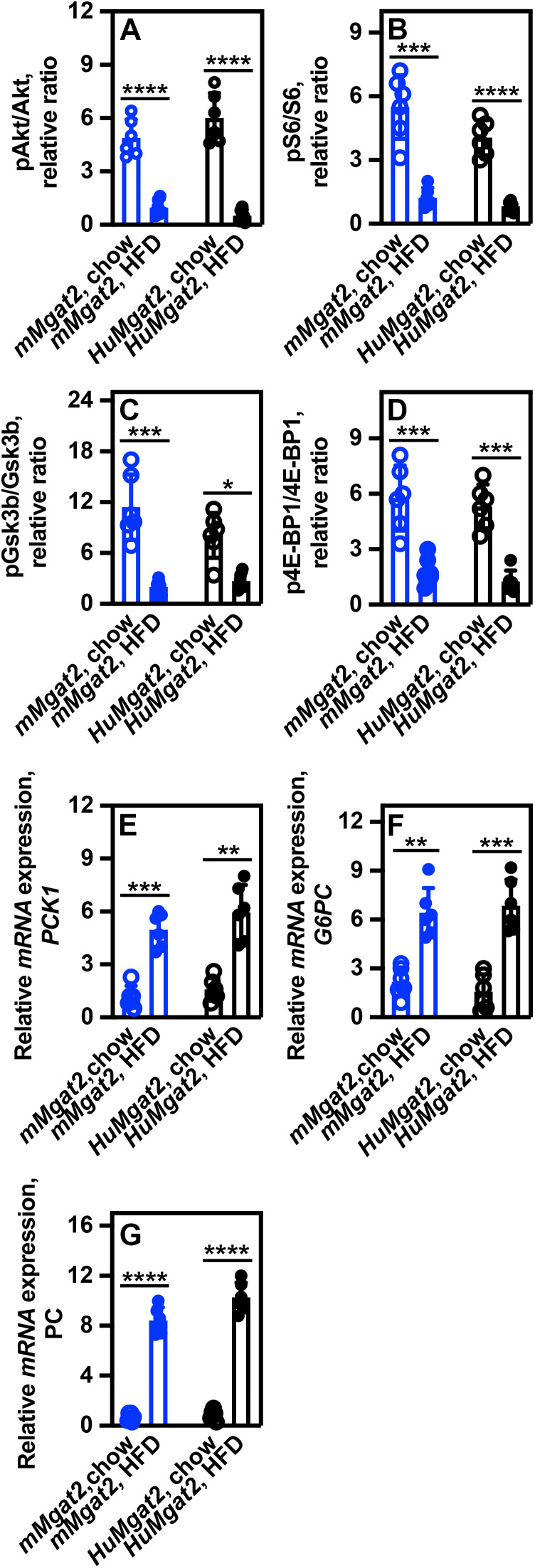
Obese *mMgat2* and *HuMgat2* mice show defects in Akt insulin signaling. Liver tissues from mMgat2 and *HuMgat2* mice were analyzed (n=6). Chow is represented by open circles, and HFD by filled circles. Protein levels were determined using E*LISA* assays. Phosphorylation rat*ios* were calculated by dividing phosphorylated protein levels by total protein levels. A) pAkt/Akt relative ratio. B) pS6/S6 relative ratio levels. C) pGsk3β/Gsk3β relative ratio. D) p4E-BP1/4E-BP1 relative ratio. mRNA expression levels were determined by *q*RT-PCR using Gapdh expression levels as a control. E) Pck1 mRNA expression levels. F) G6PC mRNA expression levels. G) PC mRNA expression levels. A two-way ANOVA analysis with Tukey's post hoc test was used for statistical analysis. Values are presented as mean ± S.D. *p<0.01; ***p<0.0001; ****p<0.00001.

Elevated pAkt^Ser473^ levels were observed in the livers of both *mMgat2* and *HuMgat2* mice fed the chow diet (Fig 2A, open circles). Levels were substantially reduced in mice that were administered the HFD (Fig 2A, closed circles). pS6^Ser235/236^ levels were higher in chow-fed mice (Fig 2B, open circles) and significantly lower in HFD-fed mice (Fig 2B, closed circles).

GSK3β phosphorylates glycogen synthase, which inhibits its activity [38]. During normal insulin signaling, GSK3β is phosphorylated on Ser9, leading to its inhibition and facilitating glucose storage as glycogen [39]. GSK3β was phosphorylated at Ser9 in both cohorts fed the chow diet but not in those fed the HFD (Fig 2C, open circles *vs.* closed circles).

The phosphorylation state of 4E-BP1, which detaches from eIF4E during insulin signaling thereby enhancing protein translation, was assessed. p4E-BP1^Thr69^ protein levels were observed in the livers of chow-fed *mMgat2* and *HuMgat2* mice and were found to be significantly lower in HFD-fed mice (Fig 2D, open circles *vs.* closed circles). These findings collectively suggest that obese *mMgat2* and *HuMgat2* mice exhibit compromised liver insulin signaling and display insulin resistance.

Impaired insulin signaling results in the inability to properly inhibit gluconeogenesis [40], which may further account for the increased basal glucose levels observed in mice fed a high-fat diet. We measured the expression levels of the gluconeogenic genes, *Pck1*, *G6pc*, and *PC*, in both chow-fed and HFD-fed mice. The expression levels of all three genes were elevated, suggesting that the defects in insulin signaling observed may have resulted in increased gluconeogenic glucose production (Fig 2E–G, open circles *vs.* closed circles).

### *mMgat2* and *HuMgat2* mice exhibit mixed hyperlipidemia

The consumption of a Western-style diet is anticipated to cause severe mixed hyperlipidemia. Therefore, we determined lipid levels in the blood and liver of *mMgat2* and *HuMgat2* mice fed two different dietary regimens.

**Fig 3:**
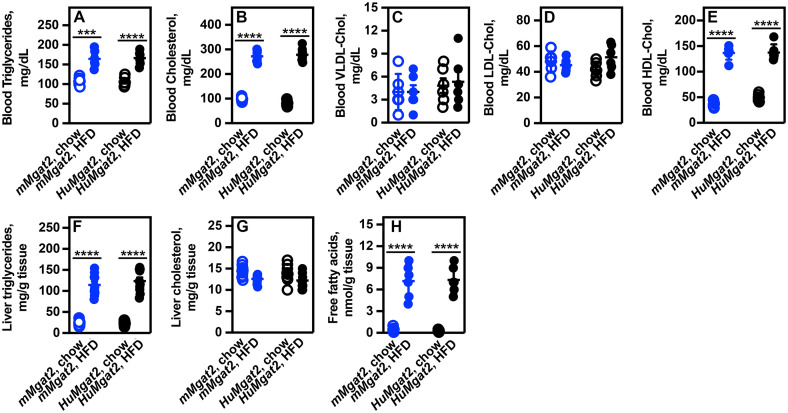
Obese *mMgat2* and *HuMgat2* mice exhibit increased blood and liver lipid levels. Mice fed either the chow diet (open circles) or the HFD (filled circles) were euthanized after 16 weeks. Blood and liver samples were collected for lipid analysis (n = 6). A, blood triglyceride levels. B, blood cholesterol levels. C, blood VLDL-Chol levels. D, blood LDL-Chol levels. E, blood HDL-Chol leve*ls*. F, liver triglyceride levels. G, liver cholesterol levels. H, free fatty acid levels. Data were analyzed using a two-way ANOVA with Dunnett’s post hoc compared to mice fed the chow diet. Data are presented as mean + S.D. ***p < 0.0001; ****p < 0.00001.

*mMgat2* and *HuMgat2* mice on a chow diet had normal triglycerides and cholesterol levels (Fig 3A, B, open circles). Both lipids increased on the HFD (Fig 3A, B, closed circles). VLDL and LDL apolipoprotein levels were unchanged on the HFD (Fig 3C, D, open circles *vs*. closed circles), but HDL levels increased ~7-fold (Fig 3H, open circles *vs*. closed circles).

Liver triglyceride levels increased in both *mMgat2* and *HuMgat2* mice that were fed the HFD (Fig 3F, open circles *vs*. closed circles), while cholesterol levels remained unchanged (Fig 3G). Free fatty acids levels also rose in HFD-fed mice (Fig 3H).

### The livers of *mMgat2* and *HuMgat2* mice exhibit elevated levels of nuclear Srebp1 and Srebp2, along with increased SREBP-dependent transcription

Srebp1 and Srebp2 are transcription factors bound to the endoplasmic reticulum that undergo cleavage in response to low cholesterol conditions [41]. Once cleaved, the soluble forms of Srebp1 and Srebp2 translocate to the nucleus, where they initiate the expression of genes involved in fatty acid and cholesterol synthesis, respectively. Elevated levels of Srebp1 and Srebp2 have been observed in obese individuals [42] and in obese mice [43]. Given the mixed hyperlipidemic phenotype of *mMgat2* and *HuMgat2* mice, we analyzed nuclear Srebp1/2 levels and their transcriptional activity in liver samples from mice on both diets.

**Fig 4:**
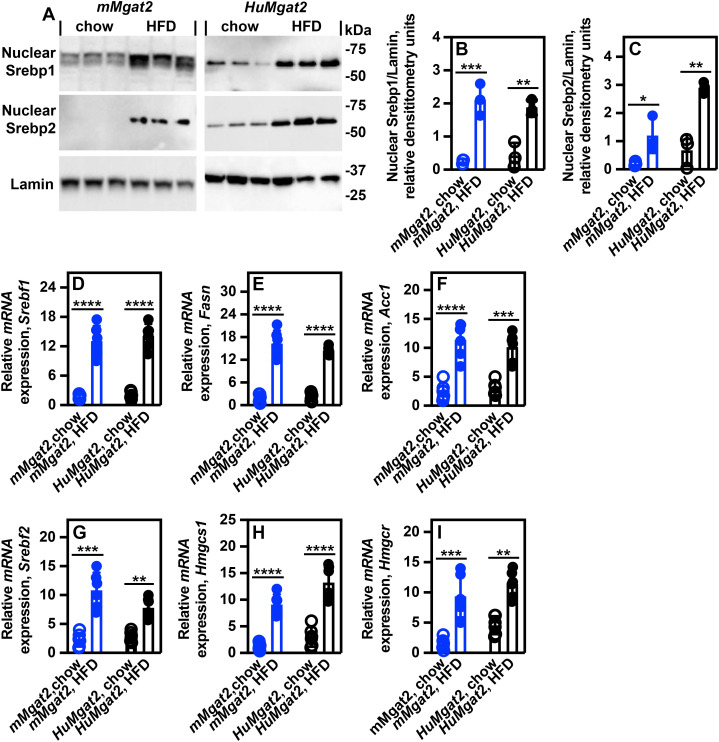
Nuclear Srebp1 and Srebp2 protein levels and Srebp-dependent lipogenic gene expression are elevated in obese *mMgat2* and *HuMgat2* mice. Total RNA and protein were extracted from the livers of *mMgat2* and *HuMgat2* mice fed chow or a HFD for *q*RT-PCR analysis and western blotting. Protein lysates were used for enrichment of nuclear Srebp1 and Srebp2 proteins as described in the Methods section (n = 3). A, western blot analysis of Srebp1 and Srebp2 protein levels. Lamin was used as a loading control. The panels in A represent a single SDS-PAGE gel that was blotted onto nitrocellulose. The single blot was used to determine Srebp1, Srebp2, and Lamin levels by stripping the blot and re-probing with the appropriate antibody. Values are the relative densitometry units using Lamin as a loading control. Relative densitometry units were calculated by dividing the raw densitometry units for Srebp1 or Srebp2 by the raw densitometry units for Lamin (n = 3). B, relative densitometry units for nuclear Srebp1. C, relative densitometry units for nuclear Srebp2. D, relative mRNA expression of *Srebf1*. E, relative mRNA expression of *Fasn*. F, relative mRNA expression of *Acc1*. G, relative mRNA expression of *Srebf2*. H, relative mRNA expression of *Hmgcs1*. I, relative mRNA expression of *Hmgcr*. Relative mRNA expression was calculated using Gapdh as an mRNA normal expression control (n = 6). A two-way ANOVA with Tukeys post hoc was used for statistical analysis. Values are mean ± S.D. *p < 0.01; **p < 0.001; ***p < 0.0001; ****p* < 0.00001*.

Elevated nuclear Srebp1 levels were observed in both HFD-fed cohorts (Fig 4A, B), correlating with higher *Srebf1*, *Fasn*, and *Acc1* gene expression (Fig 4D–F). Increased nuclear Srebp2 levels were also noted (Fig 4A, C), along with elevated *Srebf2*, *Hmgcs1*, and *Hmgcr* gene expression (Fig 4G–I).

### Obese *mMgat2* and *HuMgat2* mice on HFD have higher liver enzyme levels

The accumulation of fat in the liver results in lipotoxicity, which subsequently leads to MASLD. To evaluate liver damage in our obese mice, we conducted measurements of blood levels for liver enzymes, including alkaline phosphatase (ALP), alanine aminotransferase (ALT), and aspartate aminotransferase (AST).

**Fig 5:**
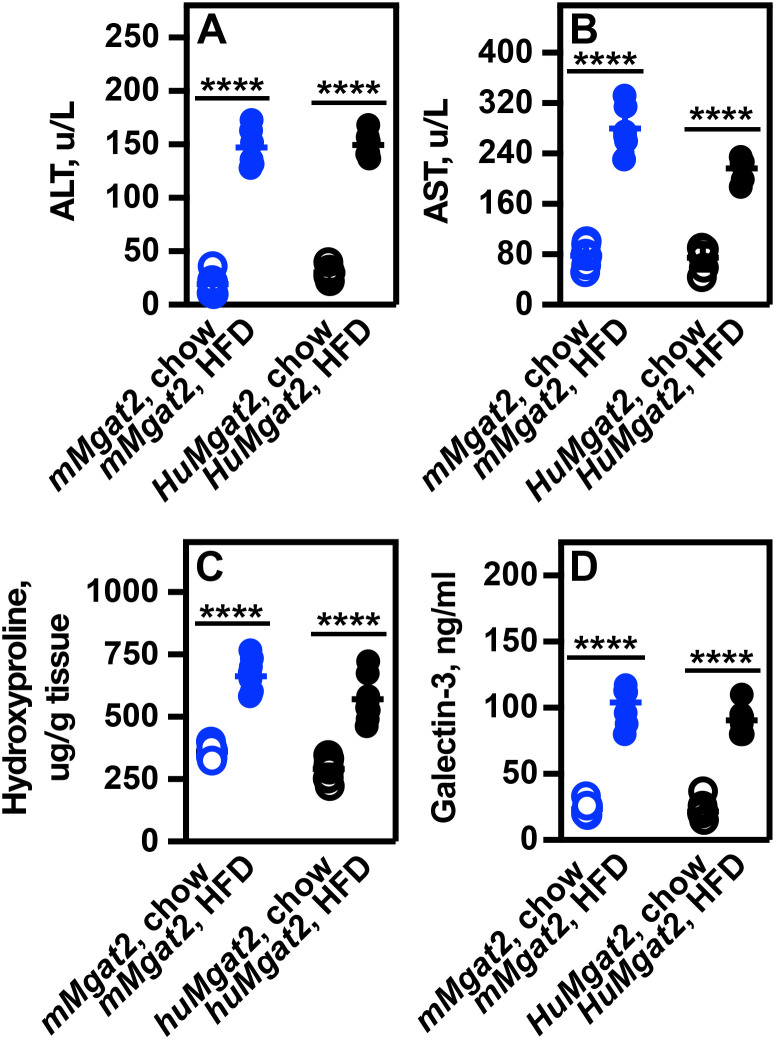
Elevated liver enzyme levels suggest hepatic damage. A, ALT blood levels. B, AST blood levels. C, Hydroxyproline levels. D, Galectin-3 levels. A two-way ANOVA with Tukeys post hoc analysis was used for statistical analysis (n = 6). Values are mean ± S.D. ****p ≤ 0.00001.

ALT levels increased by 5.6-fold, and AST levels rose by 3.2-fold (Fig 5A, B, open circles *vs*. closed circles). No increase in ALP levels was observed in either cohort (not shown).

Hydroxyproline serves as a biomarker for extracellular matrix deposition and liver damage. Both cohorts fed the HFD exhibited a 2-fold increase in hydroxyproline levels (Fig 5C). Galectin-3, which is an early marker for liver disease and MASLD, showed a 3.5 fold increase (Fig 5D).

### Obese *mMgat2* and *HuMgat2* mice fed a high-fat diet develop MASLD

Obesity is a major risk factor for the development of MASLD. Our biochemical analysis demonstrated that obese *HuMgat2* mice exhibited significant liver damage. To identify histological indicators of MASLD, we employed H&E staining to observe steatosis and inflammation, and trichrome C staining to detect fibrosis in liver tissues.

**Fig 6:**
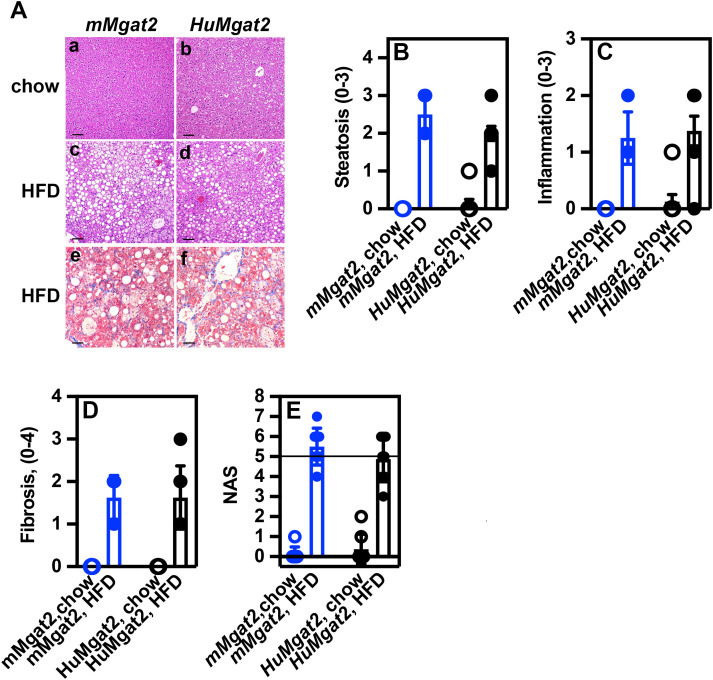
Obese *mMgat2* and *HuMgat2* mice develop MASLD and fibrosis on an HFD. A, Liver tissue sections from mice fed either the chow diet or a high-fat diet (HFD) were stained using Hemolysin & Eosin (H & E) (panels *a-d*) or Trichrome C (panels *e* & *f*) and analyzed by microscopy to detect liver damage (n = 8). A certified veterinary pathologist assessed tissue sections for steatosis, inflammation, and fibrosis. B, Average steatosis stage values. C, Average inflammation stage values. D, Average fibrosis stage values. E, A calculated NAS value was obtained by combining steatosis, inflammation, and fibrosis staging values and dividing by the number of mice per cohort [45]. Scale bar = 100 µm.

Normal hepatocyte morphology was observed in the livers of *mMgat2* and *HuMgat2* mice fed the chow diet (Fig 6A*a*, A*b*, chow). There were no indications of steatosis or macrophage infiltration based on H & E staining, nor fibrosis based on trichrome C staining. Hepatocytes from the livers of mice fed the HFD exhibited enhanced fat accumulation with both micro- and macrovesicular steatosis being observed (Fig 6A*c*, A*d*). Both cohorts showed increased inflammatory macrophage infiltration (Fig 6A*c*, A*d*). A low level of fibrosis was detected with trichrome C staining (Fig 6A*e*, A*f*). The calculated NAS for *mMgat2* and *HuMgat2* mice was 5.5 and 4.9, respectively (Fig 6E). Mice with a NAS > 5 are classified as having developed MASH [44]. *mMgat2* mice on a high-fat diet developed MASH. *HuMgat2* mice on the same diet had a NAS close to significant, with standard deviation indicating disease development. NAS is determined by summing the steatosis + inflammation + fibrosis stage scores for each individual mouse and dividing by the number of mice [45].

### High TGFβ-1 levels trigger hepatic stellate cell activation and increased liver collagen formation

Hepatic stellate cells (HSCs) are quiescent in healthy livers but become activated during the transition from MASLD to MASH. Due to the presence of fibrosis in the livers of obese *mMgat2* and *HuMgat2* mice, the levels of Tgfβ-1 were assessed. A commercial Tgfβ-1 assay kit was used to measure both its latent and active forms.

**Fig 7:**
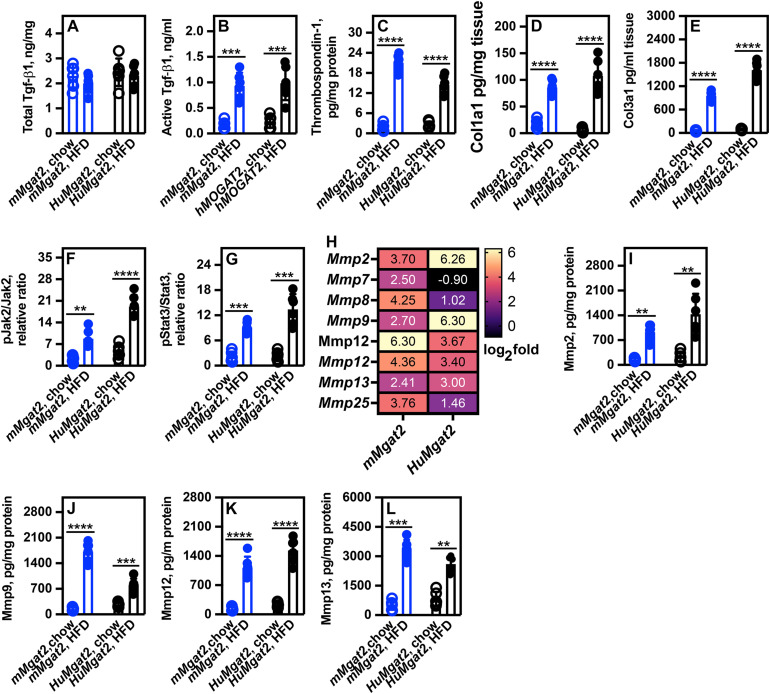
Tgfβ-1 levels drives liver collagen deposition and pJak2-pStat3 signaling in obese *mMgat2* and *HuMgat2* mice. Total Tgfβ-1 includes both latent and active forms. A commercial assay kit quantified the total and active forms of Tgfβ-1. A, total Tgf-β1 protein levels. B, total active Tgf-β1 protein levels. Protein levels were measured using ELISA assays. C, Thrompospondin-1 levels. D, Col1a1 protein levels. E, Col3a1 protein levels. Phosphorylation ratios were calculated by dividing phosphorylated protein levels by total protein levels. F, pJAK2^Tyr1007/Tyr1008^/Jak2 phosphorylation ratio. G, pStat3^705^/Stat3 phosphorylation ratio. H, Heatmap of *Mmp* gene expression data from RNASeq analysis. I, Mmp2 protein levels. J, Mmp9 protein levels. K, Mmp12 protein levels. L, Mmp13 protein levels. A two-way ANOVA with Tukeys post hoc analysis was used for statistical analysis (n = 6). Values are mean ± S.D. **p ≤ 0.001;***p ≤ 0.0001; ****p ≤ 0.00001.

Total Tgf-β1 levels remained unchanged across all feeding conditions (Fig 7A), while active Tgf-β1 levels were significantly increased in both groups fed the high-fat diet (Fig 7B).

Thrombospondin-1 activates Tgfβ-1 in hepatic stellate cells (HSCs). Although its levels are typically low in the liver, they increase in metabolic-associated steatohepatitis (MASH). Our RNA sequencing data revealed 8.6- and 11-fold increases in Thbs1 gene expression in obese *mMgat2* and *HuMgat2* mice with MASLD, respectively. Thrombospondin-1 protein concentrations were quantified using ELISA assays in the livers of mice fed either chow or the HFD.

Thrombospondin-1 protein levels were markedly elevated in the livers of obese *mMgat2* and *HuMgat2* mice compared to those maintained on a chow diet (Fig 7C, open circles *vs*. closed circles).

The activation of hepatic stellate cells (HSCs) by Tgfβ-1 enhances the secretion of type I (Col1a1) and type III (Col3a1) collagens. Elevated protein levels of Col1a1 and Col3a1 were observed in the livers of both cohorts subjected to the high-fat diet (HFD) (Fig 7D, E).

Tgfβ-1 activates Jak2-STAT3 signaling in HSCs [46], leading to matrix metalloproteinase gene expression (*Mmp*) [47–49]. The levels of pJak2^Tyr^^1007/Tyr1008^ and pStat3^Tyr705^ were elevated in the livers of obese mice compared to chow-fed mice (Fig 7F, G). RNA sequencing analysis indicated an upregulation of several *Mmp* genes in obese mice livers (Fig 7H), which corresponded with increased protein levels of Mmp2, Mmp9, Mmp12, and Mmp13 (Fig 7I–L).

### *mMgat2* and *HuMgat2* primary hepatocytes initiate pJak2-pStat3 signaling under lipotoxic conditions

The addition of palmitic acid (PA) to cells serves as a cell model for MASLD. Previous research demonstrated that treating HepG2 cells with 500 μM PA induces steatosis and increases JAK1^Tyr1022^ and JAK2^Tyr1007/Tyr1008^ levels [50]. Primary hepatocytes were isolated from *mMgat2* and *HuMgat2* mice, treated with PA, and examined for apoptosis through determining the levels of pJAK2^Tyr1007/Tyr1008^ and pStat3^Tyr705^.

**Fig 8:**
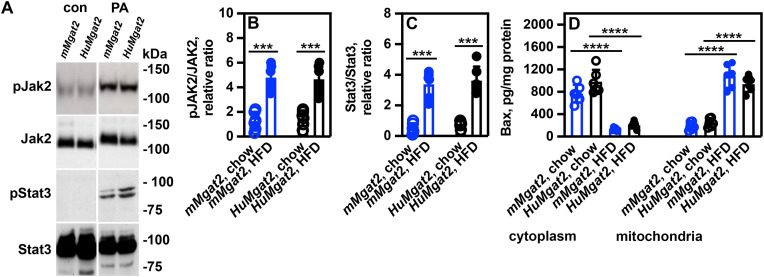
Under steatotic conditions, primary *mMgat2* and *HuMgat2* hepatocytes induce apoptosis. Primary hepatocytes were incubated with or without 500 µM PA for 24 hours. Cell lysates were then obtained for the determination of total pJAK2^Tyr1007/Tyr1008^ and pStat3^Tyr705^ levels through western analysis and ELISA. Cytoplasmic and mitochondrial fractions were isolated to measure Bax protein levels using ELISA. The panels in section A represent a single SDS-PAGE gel which was transferred onto nitrocellulose. The panels in A represent a single SDS-PAGE gel that was blotted onto nitrocellulose. This blot was used to assay pJak2, Jak2, pStat3, and Stat3 levels by sequentially stripping and re-probing with the relevant antibodies. (A), protein levels of pJak2, Jak2, pStat3, and Stat3. B, pJAK2 ^Tyr1007/Tyr1008^/JAK2 relative protein ratio (ELISA). C, pStat3^Tyr705^/Stat3 relative ratio (ELISA). D, Bax protein levels (ELISA). A two-way ANOVA with Tukeys post hoc analysis was used for statistical analysis (n = 6). Values are mean ± S.D. ***p ≤ 0.0001; ****p ≤ 0.00001.

Western analysis showed low pJAK2^Tyr1007/Tyr1008^ levels in liver protein from both cohorts fed chow (Fig 8A, chow), which significantly increased with a HFD (Fig 8A, HFD). No pStat^Tyr705^ was detected in chow-fed mice but was induced with a HFD (Fig 8A, chow vs. HFD). ELISA assays confirmed these Western results (Fig 8B, C).

The pro-apoptotic protein Bax moves from the cytoplasm to the mitochondria during apoptosis, increasing membrane permeability and cell death [51]. To confirm that PA-treated primary hepatocytes were undergoing apoptosis, we measured cytosolic and mitochondrial Bax protein levels.

In untreated *mMgat2* and *HuMgat2* hepatocytes, Bax was predominantly located in the cytoplasm Fig 8D, cytoplasm). However, under lipotoxic conditions induced by PA treatment, Bax translocated to the mitochondria (Fig 8D, mitochondria). This indicates that steatotic *mMgat2* and *HuMgat2* primary hepatocytes initiate apoptosis, like observations in the livers of mice fed a high-fat diet.

### Pathways related to lipid and cellular metabolite homeostasis exhibit alterations in mice subjected to a HFD

We conducted a comprehensive analysis and comparison of RNASeq data from four liver samples of *mMgat2* and *HuMgat2* mice subjected to either chow or high-fat diet (HFD). The gene expression levels were averaged and systematically compared between the two dietary groups.

GO ontology analysis with a log_2_ fold change >2.0 and padj<0.05 identified altered pathways in xenobiotic, organic hydroxy compounds, and sulfur metabolism in *mMgat2* and *HuMgat2* mice livers (S1 Fig). Pathways for fatty acid biosynthesis and metabolism, lipid catabolism, and steroid and cholesterol metabolism were also affected.

**Fig 9:**
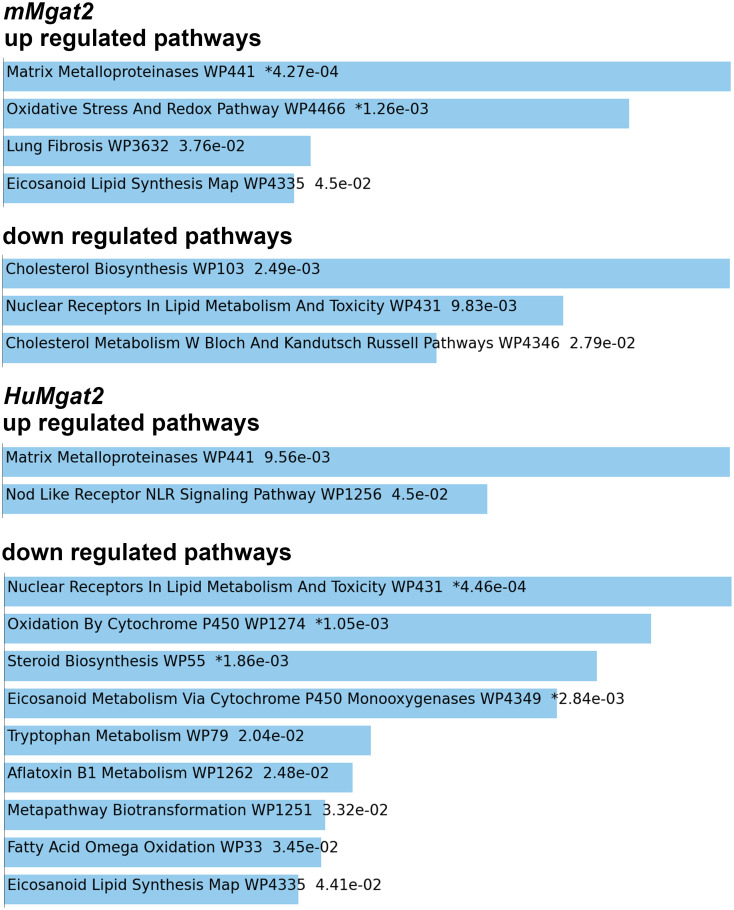
*mMgat2* and *HuMgat2* mice exhibit a transcriptomic profile indicating impairments in metabolite detoxification and lipid metabolic processes. An analysis of dysregulated genes using WikiPathway Mouse 2024 identified 307 upregulated genes and 259 downregulated genes, with a significance threshold of log2 fold change >2.5 and an adjusted p-value <0.01. These genes were used for pathway analysis. Pathways found to be dysregulated, with p-values <0.05, are listed. Gene expression levels were compared between mice fed the HFD and those fed a chow diet.

**Fig 10:**
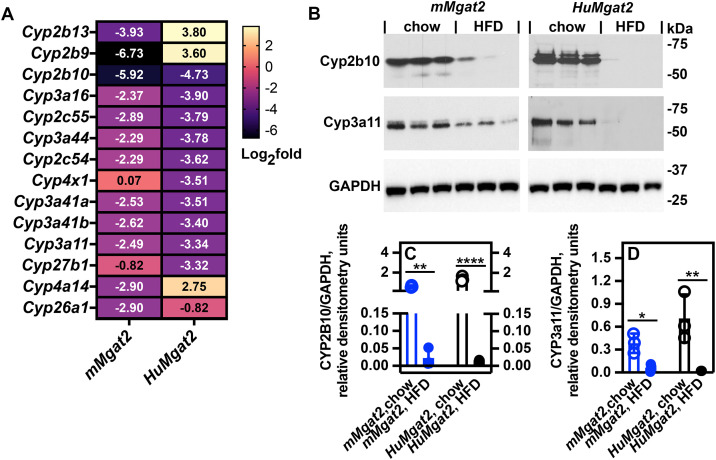
Obese *HuMgat2* mice show defects in cytochrome P450 protein levels. Liver tissue cell lysates were analyzed from mice fed either a chow diet or the HFD. The panels B represent a single SDS-PAGE gels blotted onto nitrocellulose. Cyp2b10, Cyp3a11, and Gapdh levels were determined by stripping the blot and re-probing with the appropriate antibody. Values are expressed as relative densitometry units using GAPDH as a loading control. Relative densitometry units were calculated by dividing the raw densitometry units for each protein by the raw densitometry units for Gapdh (n = 3). A, Heatmap of RNASeq expression levels of several *Cyp* genes. B, western analysis of Cyp2b10 and Cyp3a11 protein levels (n = 3). C, Relative densitometry units of Cyp2b10. D, relative densitometry units of Cyp3a11. There was one blot for the *mMgat2* samples and one blot for the *HuMgat2* samples. A two-way ANOVA with Tukeys post hoc analysis was used for statistical analysis. Values are mean ± S.D. *p ≤ 0.01;**p ≤ 0.001; ****p ≤ 0.00001.

We employed a significance threshold of log_2_ fold change >2.5 and an adjusted p-value <0.01 for pathway analysis. Our results identified 307 upregulated genes and 259 downreg*ula*ted genes. Analysis using WikiPathways mouse 2024 *wi*th these dysregulated genes highlighted pathways with p-values <0.05 that were significantly altered. Specifically, nuclear receptors involved in lipid metabolism and toxicity were found to be downregulated in both cohorts fed the high-fat diet (HFD) (Fig 9). The nuclear pregnane (Pxr) and constitutive androstane receptor (CAR) pathways, which regulate cytochrome P450 genes (Cyp) involved in detoxifying metabolites, were notably impacted. Among the top 50 genes altered in the livers of *HuMgat2* mice subjected to the HFD (S1 Table), 13 belonged to the *Cyp* gene family, including the CAR target gene, *Cyp2b10* (Fig 10A). Most *Cyp* genes exhibited downregulation, except for *Cyp2b13*, *Cyp2b9*, and *Cyp4a14*, which were upregulated.

To further investigate the RNASeq analysis, we assessed the protein expression levels of Cyp2b10 and Cyp3a11, which were anticipated to be downregulated in mice fed a high-fat diet (HFD). Western blot analysis revealed a decrease in the protein levels of both Cyp2b10 and Cyp3a11 in the livers of *mMgat2* and *HuMgat2* mice following HFD treatment (Fig 10B–D).

**Fig 11:**
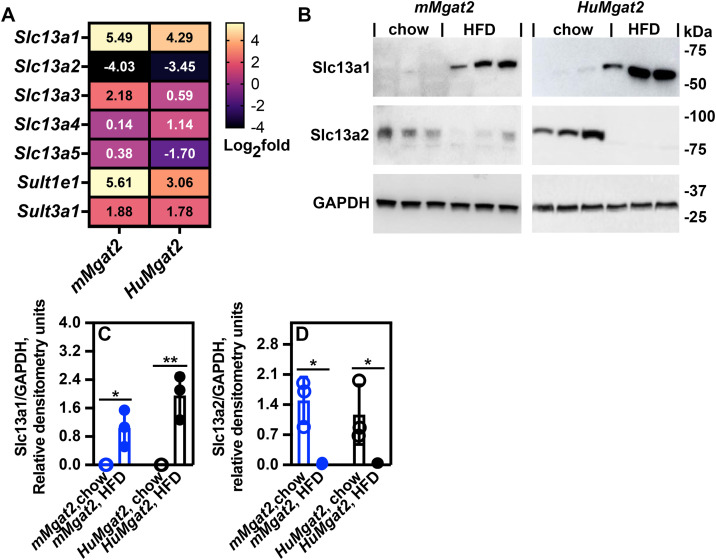
Obese *mMgat2* and *HuMgat2* mice show defects in solute transporter protein levels. A, Heatmap of RNASeq expression levels of several *Slc13a* genes. B, Slc13a1and Slc13a2 protein levels as detected by western analysis (n=3). C, relative densitometry units of Slc13a2. (D) Relative densitometry units of Slc13a2. GAPDH protein levels were used as a control for total protein. There was one blot for the *mMgat2* samples and one blot for the *HuMgat2* samples. A two-way ANOVA with Tukeys post hoc analysis was used for statistical analysis. Values are mean ± S.D. *p≤0.01; **p≤0.001.

RNASeq analyses identified changes in *Slc13a* solute transporter gene expression in response to HFD feeding (Fig 11A). The Slc13a gene family is responsible for regulating the transport of anions as well as di-carboxylic and tri-carboxylic acids. Western blot analysis did not detect the Slc13a1 protein in the livers of *mMgat2* and *HuMgat2* mice fed a chow diet (Fig 11B). However, levels of this protein were significantly increased in both groups when fed a high-fat diet (HFD) (Fig 11B, C). Conversely, Slc13a2 protein levels were highest in mice fed the chow diet and decreased in those fed the HFD (Fig 10B, D).

**Fig 12:**
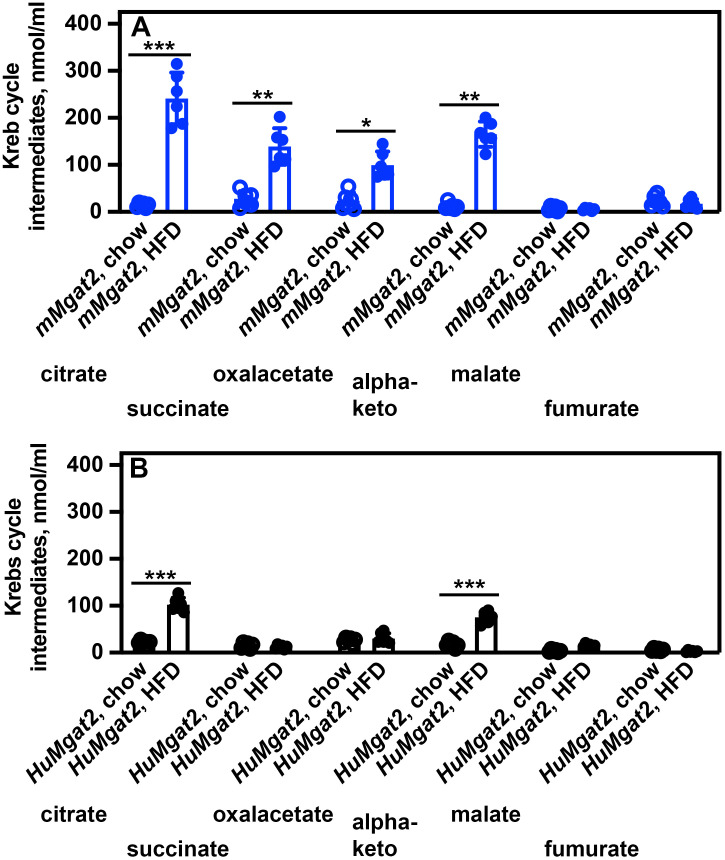
Obese *mMgat2* and *HuMgat2* mice have elevated Krebs cycle intermediate levels. Blood from *mMgat2* and *HuMgat2* was obtained at the time of euthanasia. A, Blood levels of the indicated intermediates in *mMgat2* mice (blue circles). B, Blood levels of the indicated intermediates in *HuMgat2* mice (black circles). A two-way ANOVA with Tukeys post hoc analysis was used for statistical analysis. Values are mean ± S.D. *p ≤* 0.01*; **p ≤ 0.001; ***p ≤ 0.0001.

Slc13a2 is a sodium dicarboxylate transporter with low affinity that regulates the transport of several intermediates of the Krebs cycle. We measured the blood levels of several Krebs cycle intermediates due to the irregular Slc13a2 protein levels observed. Several intermediate levels were elevated in blood from *mMgat2* mice fed the HFD (citrate, succinate, oxalacetate, and α-ketoglutarate) (Fig 12A), while only citrate and α-ketoglutarate levels were elevated in *HuMgat2* mice (Fig 12B).

## Discussion

Obesity is associated with cellular events that contribute to metabolic diseases. A diet high in fats and carbohydrates, commonly found in Western diets, is linked to obesity, hyperlipidemia, and increased LDL-cholesterol levels, which are risk factors for cardiovascular disease. This diet is also associated with hyperglycemia and insulin resistance, which are primary factors in the development of type 2 diabetes, as well as hypertension, which is related to heart disease and stroke. Additionally, hypertriglyceridemia can result in increased liver fat and the progression of MASLD/MASH.

The HFD used in our studies was the initiating factor contributing to the obesity phenotype and its associated risk factors [52]. Consequently, it played a significant role in advancing the changes observed in liver toxicity and dysfunction [53,54]. Additionally, it is established that obesity and related risk factors precede the onset of metabolic-associated fatty liver disease (MASLD) [55,56]. It is important to note that not all individuals consuming a western-style diet develop MASLD, nor do all individuals with obesity. MASLD is a multifaceted disease influenced by diet, genetic predisposition, obesity, insulin resistance, and type 2 diabetes [55–57].

*HuMgat2* mice were initially developed to test MOGAT2 inhibitors for the treatment of MASLD/MASH. Recent findings indicated that *HuMgat2* mice, when fed a steatotic diet, developed MASLD/MASH and exhibited all the characteristics of the disease observed in *mMgat2* wild-type mice [36]. We now demonstrate that *HuMgat2* mice displayed typical metabolic phenotypes associated with caloric overload due to high-fat diet consumption. Specifically, these mice became obese, hyperlipidemic, glucose intolerant, insulin resistant (pre-diabetic state), and developed MASLD and potentially MASH, like *mMgat2* mice on the same diet. The fact that *HuMgat2* mice exhibit appropriate responses to both steatotic and obesity diets underscores their value as a pre-clinical model and tool for studying the mechanisms of cell damage resulting from overeating and obesity.

RNASeq analysis revealed that pathways involved in collagen deposition and metabolite homeostasis were altered due to obesity induced by the HFD. In mice subjected to the HFD, the matrix metalloproteinase pathway exhibited upregulation, whereas lipid receptor signaling was diminished. Several *Mmp* genes were upregulated, and Mmp2/9/12/13 protein expression increased in the livers of obese mice. Elevated levels of Col1a1 and Col3a1 proteins correlated with increased collagen protein levels and fibrosis, as observed through histological staining, likely driven by Tgfβ-1-dependent activation of HSCs.

### Nuclear lipid receptor signaling was downregulated in *mMgat2* and *HuMgat2* mice fed the HFD

Cytochrome P450 *Cyp* genes exhibited significant downregulation in obese mice. RNASeq data indicated that the expression of the *Cyp2b10* gene, a target of CAR, along with the levels of Cyp3a11 protein, were reduced in these mice. This observation suggests that PXR and CAR signaling pathways were impaired [22,23]. Consequently, this may result in the accumulation of Cyp substrates such as steroids, retinoids, and ω-hydroxylated fatty acids, all of which are implicated in the pathogenesis of MASH [58].

Interestingly, levels of *Cyp2b13*, *Cyp2b9*, and *Cyp4a14* increased in livers from both cohorts. *Cyp4a14*, a murine counterpart of human Cyp4a hydroxylase, is involved in the ω-hydroxylation of MCFAs and arachidonate [59]. Elevated *Cyp4a14* expression is seen in MASLD patients, and *Cyp4a14*^*-/-*^ mice are resistant to MASLD and fibrosis [60].

Protein levels of the Slc13a solute transporter family, specifically Slc13a1 and Slc13a2, were altered in both cohorts fed the HFD. Slc13a2 is responsible for transporting carboxylic acids such as citrate and succinate [61]. *Slc13a2*^*-/-*^ mice exhibit elevated secretion of TCA cycle intermediates in urine, indicating impaired cellular uptake of carboxylic acids. Alterations in citrate levels have been linked to various cancers, whereas succinate accumulation initiates the innate immune response [62]. Obese *db*/*db* mice secrete high levels of TCA intermediates, including succinate, citrate, malate, and aconitate [11]. Increased Kreb cycle intermediates in the serum of obese mice were observed.

Slc13a proteins have been identified as targets for the discovery of small molecule inhibitors. *Slc13a5*^*-/-*^ mice exhibit resistance to obesity [63]. These mice show a higher energy expenditure rate, enhanced mitochondrial biogenesis and fatty acid β-oxidation, and decreased hepatic lipogenesis. Elevated levels of *Slc13a5* are found in MASLD patients [64]. *Slc13a5* is involved in extracellular citrate uptake [62] and is considered a target for treating metabolic diseases, with various inhibitors being tested [65–67].

What are the effects of improper cell metabolite detoxification? In obese mice, changes in *Cyp* gene expression and protein levels may lead to the accumulation of lipids and xenobiotics. For example, arachidonate can produce eicosanoids [68], while retinoids and retinyl esters are associated with MASH [69]. Alterations in solute carrier transporter levels can negatively affect various biochemical and signaling mechanisms. The accumulation of TCA intermediates may disrupt mitochondrial function, energy production, and redox balance by changing the NADH/NAD+ ratio, electron transport chain functionality, and several cell signaling processes.

Sulfotransferases, such as *Sult1e1*, which show increased expression levels in obese mice, catalyze the sulfonation of various compounds including steroids and bile acids [70]. Studies have demonstrated that the ablation of *Sult1e1* reduces the inflammatory response caused by LPS-induced sepsis through the inactivation of estrogen, an anti-inflammatory hormone [71]. We observed a 6.2-fold and 2.7-fold increase in *Sult2b1* sulfotransferase gene expression in obese *mMgat2* and *HuMgat2* mice, respectively. Cholesterol, a substrate of *Sult2b1*, forms cholesterol sulfate, which subsequently activates protein kinase C and mitogenic transcription factors [72]. There are mechanistic links between reduced sulfate transport in rat kidneys, metabolic acidosis, and cell death [73].

Disruption of metabolite homeostasis has significant consequences for obesity-related metabolic diseases. Further research into restoring cellular metabolite homeostasis may identify new therapeutic targets for managing diseases linked to high caloric intake from fats and carbohydrates, which contribute to obesity.

## Conclusion

Excessive caloric intake leads to obesity, which is associated with various metabolic diseases. When subjected to a HFD, *mMgat2* and *HuMgat2* mice displayed obesity, glucose intolerance, insulin resistance, and MASLD, and most likely MASH. These metabolic disorders were linked to defects in insulin signaling and excessive lipid accumulation in their livers. Furthermore, multiple pathways involved in cellular detoxification, such as those regulating the expression of *Cyp*, *Slc13a*, and *Sult* genes and proteins, were found to be dysregulated, likely resulting in the buildup of toxic metabolites within cells, thereby exacerbating disease progression and leading to cell death. It should be noted that one limitation of our study is that only male mice were tested. Additio*nally,* further metabolomics analysis is required to identify the specific toxic metabolites accumulating in the livers of *HuMgat2* mice.

## Methods

### Miscellaneous reagents

Chemicals were purchased from Millipore Sigma (St. Louis, MO.). Proteinase inhibitor-phosphatase inhibitor cocktail (#7834) and nuclease (#88701) were purchased from Millipore Sigma (St. Louis, MO.). Collagenase (#C3867) was purchased from Millipore Sigma (St. Louis, MO.). All SDS-PAGE and western supplies were purchased from BIO-RAD (Philadelphia, PA.). Reagents for histological staining were purchased from Agilent (Santa Clara, CA.). Primers and master mix used for *q*RT-PCR were purchased from Thermo Fisher Scientific (Carlsbad, CA.).

### Generation of *HuMgat2 and mMgat2* mice

*HuMgat2* mice were created in the C57BL/6J background by Cyagen (Santa Clara, CA) as previously described [36]. The mouse *MGAT2* locus was replaced with human *MOGAT2* cDNA without introns. The correct sequence was confirmed by restriction digestion and PCR before generating the *HuMgat2* mouse [36]. Human *MOGAT2* expression was driven by the mouse *MGAT2* promoter, resulting in comparable mRNA and protein levels to mouse MGAT2, validated by qRT-PCR and western analysis [36]. All animal studies were approved by Invivotek’s IACUC (#97258) and followed the “Institutional Animal Care and Use Committee Handbook.”

### Metabolic feeding studies

8 weeks old male *mMgat2* and *HuMgat2* mice were administered either a chow diet (Purina, Picochow 5053, Lab Diets; 24% of calories from protein, 63% calories from carbohydrate, 13% calories from fat) or a high-fat diet (Research Diets, New Brunswick, NJ, #RD12079B (Western Diet); 13% calories from protein. 49% calories from carbohydrate (23% from sucrose), 40% calories from fat, 0.02% calories from cholesterol) for a duration of 16 weeks (n = 8). Oral glucose tolerance tests were conducted at week 12, followed by insulin tolerance tests at week 14. The mice were subsequently euthanized after completing the 16-week period. The mice were not fasted prior to being euthanized. Mice were euthanized by CO_2_-induced asphyxiation, followed by cervical dislocation.

All procedures were approved by the Genesis Drug Discovery and Development Institutional Care and Use Committee and performed in accordance with institutional SOPs and AUPs. The experimental design incorporated the principles of Replacement, Reduction, and Refinement and every effort was made to minimize suffering and distress to ensure the ethical and humane treatment of animals. All mice were checked daily, and any moribund animals were sacrificed. All mouse studies were conducted by Invivotek, L.L.C. Liver and blood samples were supplied for analysis. Histological staining was performed by Medical Diagnostics Laboratories, L.L.C. Histological staging was performed by a licensed veterinary pathologist.

### Oral glucose tolerance test and insulin measurements

Mice were fasted for 16 hours before the study. Blood glucose levels were measured at baseline, 15, 30, 60, and 120 minutes after receiving 2g/kg of 100 mg/ml glucose by oral gavage. Blood samples taken were also used to measure plasma insulin levels. Glucose was measured with a One-touch Ultra 2 glucometer, and insulin with an electrochemiluminescence kit (MA2400 Mouse/Rat insulin kit K152BZC, Meso Scale Discovery).

### Insulin tolerance test

Mice were fasted for 4 hours before the study initiation. Baseline insulin levels were obtained from a tail cut (distal 2 mm of the tail). Mice on a chow diet received 0.5 units/kg of insulin via intraperitoneal injection, while those on a HFD received 1.0 units/kg of insulin. Blood glucose levels were measured at 15, 30, 60, and 90 minutes.

### Serum clinical chemistries

Terminal serum samples were collected and analyzed using an ACE Alera (Alfa Wasserman) according to the manufacturer’s protocol.

### Lipid extraction from mouse tissues

100 mg of liver tissue was homogenized and extracted using hexane:2-propanol (3:2). Samples were spun, transferred to glass tubes, and washed with 0.9% NaCl. After centrifugation, the aqueous phase was removed, and the organic phase was dried and stored in isopropyl alcohol until use. Triglycerides and cholesterol levels were measured using the Cayman Triglyceride Colorimetric Assay kit and the Promega Cholesterol/Cholesterol Ester GloTM assay kit, respectively. Apolipoprotein levels were determined using the LipoPrint LDL system (Quantimetrix, Redondo Beach, CA) per the manufacturers protocol.

### Protein extraction

Tissue samples were homogenized in RIPA buffer with phosphatase and protease inhibitors. Cell lysates were obtained via low-speed centrifugation and stored at −20°C until use. Protein concentrations were determined using the Pierce™ BCA Protein Assay Kit.

### Nuclear isolation

Nuclear fractions were isolated from tissue samples using the NE-PERTM Nuclear and Cytoplasmic Extraction kit (ThermoFisher Scientific, #78833). Lamin was used as a nuclear marker.

### Cytoplasm and mitochondrial isolation from primary hepatocytes

Cytoplasmic and mitochondrial fractions were isolated with a commercial kit (Abcam, #ab109719). GAPDH served as a cytoplasmic control, and Hsp70 was the mitochondrial marker.

### Western blotting

Cell lysates containing 25 μg of protein were resuspended in sample buffer and were resolved by SDS-PAGE. After transfer to nitrocellulose, membranes were incubated for 1 hr to overnight with TBST (Tris-buffered saline, 0.1% Tween 20) containing 10% milk.

The membranes were washed several times with TBST before being incubated with primary antibodies for 12–16 hours. Subsequently, the membranes were washed several times with TBST and were then incubated with secondary antibodies for 1–4 hours. Following additional washes with TBST, the membranes were treated with a chemiluminescent agent (Cytiva, Amersham ECL Prime Western Blotting Detection Reagent, Wilmington, DE). Protein detection was carried out using an Amersham Imager 600. GAPDH was used as the loading control for all western blot analyses.

Each western blot panel represents a single SDS-PAGE gel. For each blot, the protein of interest was detected, the blot was stripped, and GAPDH was detected. In cases involving the detection of phospho-forms of a protein, phospho-proteins were detected, the blot was stripped, followed by detection of the total protein, and then stripped again for subsequent GAPDH control detection.

For densitometry analysis, all TIFF images of western blots were adjusted to 150 brightness using the Image tab/adjustments/brightness function within Adobe Photoshop (version 26.1.0) to achieve a normalized baseline.

All antibodies used are listed in S1 Table.

### ELISA assays

Elisa assays were performed as per manufacturer’s instructions using liver tissue lysates. Assay kits used are listed in S2 Table.

### Hydroxyproline assay

Hydroxyproline levels were determined as described using liver tissue [74].

### Galectin-3 assay

Galectin-3 levels were determined from serum by ELISA.

### Krebs cycle intermediate assays

Krebs cycle intermediate levels in blood were measured using plasma. The assay kits used included Citrate (Abcam, #ab83396), Succinate (Abcam, #204718), Oxalacetate (Abnova, #KA3793), α-Ketoglutarate (Abcam, #83431), Malate (Abcam, #ab83391), and Fumarate (Abcam, #102516) kits. Validation was done by comparing samples from each cohort with LC-MS analysis [10].

### Cell based bioassay for TGF-β1 activity

A commercial assay kit was used to determine Τgfβ-1 levels according to the manufacturer’s protocol (BPS Bioscience, San Diego, CA, Cat# 60544).

### Histology

Liver tissues embedded in parafilm were sectioned, mounted onto slides, then dehydrated and delipidated before being stained with hematoxylin and eosin (Leica Biosystems) or trichrome C (Abcam).

### Isolation of primary hepatocytes from *mMgat2* and *HuMgat2* livers

Primary mouse hepatocytes were isolated from livers as described [75]. Briefly, primary hepatocytes were isolated by *vena cava* cannulation. Livers were perfused with EDTA to remove blood followed by collagenase perfusion. Livers were dissected and primary hepatocytes were obtained by low speed centrifugation and subsequent plating.

### Palmitic acid treatment of primary mouse hepatocytes

Cells grown in DMEM containing 5% BSA were treated with 500μM palmitic acid (PA) for 24 hr. Cells were collected by gentle scraping off plates. Cells were incubated with lysis buffer (50mM Tris, pH 8.2, containing 8M Urea, 75 mM NaCl, proteinase and phosphatase inhibitors plus nuclease) at 4^o^C for 2 hr. Lysed cells were then centrifuged at 12,000 X g and supernatants were used for protein determination.

### *q*RT-PCR analysis

Total RNA was extracted from liver tissue using the RNeasy Plus Universal kit (Qiagen, Germantown, MD) and treated with RNase-free DNase. RNA was reverse transcribed with the QuantiTect Reverse Transcription kit (Qiagen, Germantown, MD) and amplified by PCR using the Power SYBR RNA-to-CT 1-step kit (ThermoFisher Scientific, Waltham, MA). Results were expressed as log_2_ fold change comparing mice fed the CDAA-HFD to those fed the chow diet.

### RNASeq analysis

Cell extracts from 15 mg of liver tissue were used to isolate RNA for RNASeq analysis using the RNeasy Plus Universal kit (Qiagen, Germantown, MD). RNA libraries were sequenced, and individual reads were aligned to the Ensembl Mus mm39 musculus genome using STAR 2.7 [76]. Differential gene expression analysis was performed with DEseq2 (v 1.26.0) [77]. A Benjamini and Hochberg adjusted p ≤ 0.05 (5% False Discovery Rate) score for a gene was regarded as it being statistically significantly different. Gene Ontology Enrichment pathways were identified using the cluster Profiler package for R [78].

### Statistical analysis

*in vivo* data were analyzed using a two-way ANOVA analysis with Dunnett’s post hoc test compared to chow-fed mice (n = 8). Data is presented as mean ± S.D. All other data were analyzed by two-way ANOVA with Tukeys post hoc analysis. All data is presented as mean ± S.D.

## Supporting information

S1 Table Antibodies used for western analysis. The source, catalog number, and dilution ratio, used for each antibody are listed.
(DOCX)

S2 Table ELISA kits used for protein analysis. The source and catalog number for each ELISA kit are listed.
(DOCX)

S1 Fig GO ontology analyses of RNASeq data of livers from *mMgat2* and *HuMgat2* mice. The log_2_fold value for each gene from mice fed the HFD was compared to the log_2_fold expression value in livers from mice fed the chow diet.
(TIF)

S2 Fig Full-length western blots. Full-length western blots used to construct western figure panels.
(PDF)

S1 File Supplemental full length westerns. (PDF)

## Abbreviations

MASLD: metabolic dysfunction-associated steatotic liver disease
MASH: metabolic dysfunction-associated steatohepatitis
OGTT: oral glucose tolerance test
ITT: insulin tolerance test
TGF-β1: transforming growth factor-β1
HSCs: hepatic stellate cells
Col1a1: collagen type I α1 chain
Col3a1: collagen type III α1 chain
PXR: pregnane X receptor
CAR: constitutive aldrostane receptor
MCFAs: medium chain fatty acids

## References

[1] Boutari C, Mantzoros CS. A 2022 update on the epidemiology of obesity and a call to action: as its twin COVID-19 pandemic appears to be receding, the obesity and dysmetabolism pandemic continues to rage on. Metabolism. 2022;133:155217. doi: 10.1016/j.metabol.2022.155217 35584732 PMC9107388

[2] Fruh SM. Obesity: risk factors, complications, and strategies for sustainable long-term weight management. J Am Assoc Nurse Pract. 2017;29(S1):S3–14. doi: 10.1002/2327-6924.12510 29024553 PMC6088226

[3] Stuart B, Lloyd J, Zhao L, Kamal-Bahl S. Obesity, disease burden, and prescription spending by community-dwelling medicare beneficiaries. Curr Med Res Opin. 2008;24(8):2377–87. doi: 10.1185/03007990802262275 18616864

[4] Lister NB, Baur LA, Felix JF, Hill AJ, Marcus C, Reinehr T, et al. Child and adolescent obesity. Nat Rev Dis Primers. 2023;9(1):24. doi: 10.1038/s41572-023-00435-4 37202378

[5] Ottosson F, Smith E, Ericson U, Brunkwall L, Orho-Melander M, Di Somma S, et al. Metabolome-defined obesity and the risk of future type 2 diabetes and mortality. Diabetes Care. 2022;45(5):1260–7. doi: 10.2337/dc21-2402 35287165 PMC9174969

[6] Ho JE, Larson MG, Ghorbani A, Cheng S, Chen M-H, Keyes M, et al. Metabolomic profiles of body mass index in the framingham heart study reveal distinct cardiometabolic phenotypes. PLoS One. 2016;11(2):e0148361. doi: 10.1371/journal.pone.0148361 26863521 PMC4749349

[7] Yin X, Chan LS, Bose D, Jackson AU, VandeHaar P, Locke AE, et al. Genome-wide association studies of metabolites in Finnish men identify disease-relevant loci. Nat Commun. 2022;13(1):1644. doi: 10.1038/s41467-022-29143-5 35347128 PMC8960770

[8] Milhem F, Komarnytsky S. Progression to Obesity: Variations in Patterns of Metabolic Fluxes, Fat Accumulation, and Gastrointestinal Responses. Metabolites. 2023;13(9):1016. doi: 10.3390/metabo13091016 37755296 PMC10535155

[9] Vanweert F, Schrauwen P, Phielix E. Role of branched-chain amino acid metabolism in the pathogenesis of obesity and type 2 diabetes-related metabolic disturbances BCAA metabolism in type 2 diabetes. Nutr Diabetes. 2022;12(1):35. doi: 10.1038/s41387-022-00213-3 35931683 PMC9356071

[10] Serena C, Ceperuelo-Mallafré V, Keiran N, Queipo-Ortuño MI, Bernal R, Gomez-Huelgas R, et al. Elevated circulating levels of succinate in human obesity are linked to specific gut microbiota. ISME J. 2018;12(7):1642–57. doi: 10.1038/s41396-018-0068-2 29434314 PMC6018807

[11] Li M, Wang X, Aa J, Qin W, Zha W, Ge Y, et al. GC/TOFMS analysis of metabolites in serum and urine reveals metabolic perturbation of TCA cycle in db/db mice involved in diabetic nephropathy. Am J Physiol Renal Physiol. 2013;304(11):F1317–24. doi: 10.1152/ajprenal.00536.2012 23467425

[12] Jitrapakdee S, St Maurice M, Rayment I, Cleland WW, Wallace JC, Attwood PV. Structure, mechanism and regulation of pyruvate carboxylase. Biochem J. 2008;413(3):369–87. doi: 10.1042/BJ20080709 18613815 PMC2859305

[13] Akhtar MJ, Khan SA, Kumar B, Chawla P, Bhatia R, Singh K. Role of sodium dependent SLC13 transporter inhibitors in various metabolic disorders. Mol Cell Biochem. 2023;478(8):1669–87. doi: 10.1007/s11010-022-04618-7 36495372

[14] NICE. Evidence reviews for the effectiveness of different diets in achieving and maintaining weight loss: Overweight and obesity management: preventing, assessing and managing overweight and obesity: Evidence review F. London: NICE Evidence Reviews Collection; 2025. 40029958

[15] Astrup A, Raben A, Geiker N. The role of higher protein diets in weight control and obesity-related comorbidities. Int J Obes (Lond). 2015;39(5):721–6. doi: 10.1038/ijo.2014.216 25540980 PMC4424378

[16] Thackrey E, Chen J, Martino C-R, Preda V. The effects of diet on weight and metabolic outcomes in patients with double diabetes: a systematic review. Nutrition. 2022;94:111536. doi: 10.1016/j.nut.2021.111536 34936947

[17] Esko T, Hirschhorn JN, Feldman HA, Hsu Y-HH, Deik AA, Clish CB, et al. Metabolomic profiles as reliable biomarkers of dietary composition. Am J Clin Nutr. 2017;105(3):547–54. doi: 10.3945/ajcn.116.144428 28077380 PMC5320413

[18] Guertin KA, Moore SC, Sampson JN, Huang W-Y, Xiao Q, Stolzenberg-Solomon RZ, et al. Metabolomics in nutritional epidemiology: identifying metabolites associated with diet and quantifying their potential to uncover diet-disease relations in populations. Am J Clin Nutr. 2014;100(1):208–17. doi: 10.3945/ajcn.113.078758 24740205 PMC4144099

[19] Wang W, Liu Y, Li Y, Luo B, Lin Z, Chen K, et al. Dietary patterns and cardiometabolic health: clinical evidence and mechanism. MedComm (2020). 2023;4(1):e212. doi: 10.1002/mco2.212 36776765 PMC9899878

[20] Kim H, Rebholz CM. Metabolomic biomarkers of healthy dietary patterns and cardiovascular outcomes. Curr Atheroscler Rep. 2021;23(6):26. doi: 10.1007/s11883-021-00921-8 33782776

[21] Rafiq T, Azab SM, Teo KK, Thabane L, Anand SS, Morrison KM, et al. Nutritional metabolomics and the classification of dietary biomarker candidates: a critical review. Adv Nutr. 2021;12(6):2333–57. doi: 10.1093/advances/nmab054 34015815 PMC8634495

[22] Oladimeji PO, Chen T. PXR: more than just a master xenobiotic receptor. Mol Pharmacol. 2018;93(2):119–27. doi: 10.1124/mol.117.110155 29113993 PMC5767680

[23] Yan J, Chen B, Lu J, Xie W. Deciphering the roles of the constitutive androstane receptor in energy metabolism. Acta Pharmacol Sin. 2015;36(1):62–70. doi: 10.1038/aps.2014.102 25500869 PMC4571311

[24] Ashibe B, Nakajima Y, Fukui Y, Motojima K. PPARα as a transcriptional regulator for detoxification of plant diet-derived unfavorable compounds. PPAR Res. 2012;2012:814945. doi: 10.1155/2012/814945 22577367 PMC3345252

[25] Eccles JA, Baldwin WS. Detoxification cytochrome P450s (CYPs) in families 1-3 produce functional oxylipins from polyunsaturated fatty acids. Cells. 2022;12(1):82. doi: 10.3390/cells12010082 36611876 PMC9818454

[26] Kotlyar M, Carson SW. Effects of obesity on the cytochrome P450 enzyme system. Int J Clin Pharmacol Ther. 1999;37(1):8–19. 10027478

[27] Jovanović M, Kovačević S, Brkljačić J, Djordjevic A. Oxidative stress linking obesity and cancer: is obesity a “radical trigger” to cancer? Int J Mol Sci. 2023;24(9):8452. doi: 10.3390/ijms24098452 37176160 PMC10179114

[28] Tan HC, Hsu JW, Tai ES, Chacko S, Wu V, Lee CF, et al. De novo glycine synthesis is reduced in adults with morbid obesity and increases following bariatric surgery. Front Endocrinol (Lausanne). 2022;13:900343. doi: 10.3389/fendo.2022.900343 35757406 PMC9219591

[29] Xia Y, Zhai X, Qiu Y, Lu X, Jiao Y. The Nrf2 in obesity: a friend or foe? Antioxidants (Basel). 2022;11(10):2067. doi: 10.3390/antiox11102067 36290791 PMC9598341

[30] Coleman RA, Lee DP. Enzymes of triacylglycerol synthesis and their regulation. Prog Lipid Res. 2004;43(2):134–76. doi: 10.1016/s0163-7827(03)00051-1 14654091

[31] Turkish AR, Henneberry AL, Cromley D, Padamsee M, Oelkers P, Bazzi H, et al. Identification of two novel human acyl-CoA wax alcohol acyltransferases: members of the diacylglycerol acyltransferase 2 (DGAT2) gene superfamily. J Biol Chem. 2005;280(15):14755–64. doi: 10.1074/jbc.M500025200 15671038

[32] Yen C-LE, Farese RV Jr. MGAT2, a monoacylglycerol acyltransferase expressed in the small intestine. J Biol Chem. 2003;278(20):18532–7. doi: 10.1074/jbc.M301633200 12621063

[33] Yen C-LE, Cheong M-L, Grueter C, Zhou P, Moriwaki J, Wong JS, et al. Deficiency of the intestinal enzyme acyl CoA: monoacylglycerol acyltransferase-2 protects mice from metabolic disorders induced by high-fat feeding. Nat Med. 2009;15(4):442–6. doi: 10.1038/nm.1937 19287392 PMC2786494

[34] Gao Y, Nelson DW, Banh T, Yen M-I, Yen C-LE. Intestine-specific expression of MOGAT2 partially restores metabolic efficiency in Mogat2-deficient mice. J Lipid Res. 2013;54(6):1644–52. doi: 10.1194/jlr.M035493 23536640 PMC3646465

[35] Banh T, Nelson DW, Gao Y, Huang T-N, Yen M-I, Yen C-LE. Adult-onset deficiency of acyl CoA:monoacylglycerol acyltransferase 2 protects mice from diet-induced obesity and glucose intolerance. J Lipid Res. 2015;56(2):379–89. doi: 10.1194/jlr.M055228 25535286 PMC4306691

[36] Corbalan JJ, Jagadeesan P, Frietze KK, Taylor R, Gao GL, Gallagher G, et al. Humanized monoacylglycerol acyltransferase 2 mice develop metabolic dysfunction-associated steatohepatitis. J Lipid Res. 2024;65(12):100695. doi: 10.1016/j.jlr.2024.100695 39505262 PMC11648239

[37] Boucher J, Kleinridders A, Kahn CR. Insulin receptor signaling in normal and insulin-resistant states. Cold Spring Harb Perspect Biol. 2014;6(1):a009191. doi: 10.1101/cshperspect.a009191 24384568 PMC3941218

[38] Beurel E, Grieco SF, Jope RS. Glycogen synthase kinase-3 (GSK3): regulation, actions, and diseases. Pharmacol Ther. 2015;148:114–31. doi: 10.1016/j.pharmthera.2014.11.016 25435019 PMC4340754

[39] Fang X, Yu SX, Lu Y, Bast RC Jr, Woodgett JR, Mills GB. Phosphorylation and inactivation of glycogen synthase kinase 3 by protein kinase A. Proc Natl Acad Sci U S A. 2000;97(22):11960–5. doi: 10.1073/pnas.220413597 11035810 PMC17277

[40] Hatting M, Tavares CDJ, Sharabi K, Rines AK, Puigserver P. Insulin regulation of gluconeogenesis. Ann N Y Acad Sci. 2018;1411(1):21–35. doi: 10.1111/nyas.13435 28868790 PMC5927596

[41] Brown MS, Goldstein JL. The SREBP pathway: regulation of cholesterol metabolism by proteolysis of a membrane-bound transcription factor. Cell. 1997;89(3):331–40. doi: 10.1016/s0092-8674(00)80213-5 9150132

[42] Pettinelli P, Del Pozo T, Araya J, Rodrigo R, Araya AV, Smok G, et al. Enhancement in liver SREBP-1c/PPAR-alpha ratio and steatosis in obese patients: correlations with insulin resistance and n-3 long-chain polyunsaturated fatty acid depletion. Biochim Biophys Acta. 2009;1792(11):1080–6. doi: 10.1016/j.bbadis.2009.08.015 19733654

[43] Shimomura I, Bashmakov Y, Horton JD. Increased levels of nuclear SREBP-1c associated with fatty livers in two mouse models of diabetes mellitus. J Biol Chem. 1999;274(42):30028–32. doi: 10.1074/jbc.274.42.30028 10514488

[44] Kleiner DE, Brunt EM, Van Natta M, Behling C, Contos MJ, Cummings OW, et al. Design and validation of a histological scoring system for nonalcoholic fatty liver disease. Hepatology. 2005;41(6):1313–21. doi: 10.1002/hep.20701 15915461

[45] Brunt EM, Kleiner DE, Wilson LA, Belt P, Neuschwander-Tetri BA NASH Clinical Research Network (CRN). Nonalcoholic fatty liver disease (NAFLD) activity score and the histopathologic diagnosis in NAFLD: distinct clinicopathologic meanings. Hepatology. 2011;53(3):810–20. doi: 10.1002/hep.24127 21319198 PMC3079483

[46] Deng Z, Fan T, Xiao C, Tian H, Zheng Y, Li C, et al. TGF-β signaling in health, disease, and therapeutics. Signal Transduct Target Ther. 2024;9(1):61. doi: 10.1038/s41392-024-01764-w 38514615 PMC10958066

[47] Xuan L, Han F, Gong L, Lv Y, Wan Z, Liu H, et al. Ceramide induces MMP-9 expression through JAK2/STAT3 pathway in airway epithelium. Lipids Health Dis. 2020;19(1):196. doi: 10.1186/s12944-020-01373-w 32829707 PMC7444274

[48] Kothari P, Pestana R, Mesraoua R, Elchaki R, Khan KMF, Dannenberg AJ, et al. IL-6-mediated induction of matrix metalloproteinase-9 is modulated by JAK-dependent IL-10 expression in macrophages. J Immunol. 2014;192(1):349–57. doi: 10.4049/jimmunol.1301906 24285838 PMC3872272

[49] Ghosh A, Pechota A, Coleman D, Upchurch GR Jr, Eliason JL. Cigarette smoke-induced MMP2 and MMP9 secretion from aortic vascular smooth cells is mediated via the Jak/Stat pathway. Hum Pathol. 2015;46(2):284–94. doi: 10.1016/j.humpath.2014.11.003 25537973

[50] Frietze KK, Brown AM, Das D, Franks RG, Cunningham JL, Hayward M, et al. Lipotoxicity reduces DDX58/Rig-1 expression and activity leading to impaired autophagy and cell death. Autophagy. 2022;18(1):142–60. doi: 10.1080/15548627.2021.1920818 33966599 PMC8865291

[51] Lopez A, Reyna DE, Gitego N, Kopp F, Zhou H, Miranda-Roman MA, et al. Co-targeting of BAX and BCL-XL proteins broadly overcomes resistance to apoptosis in cancer. Nat Commun. 2022;13(1):1199. doi: 10.1038/s41467-022-28741-7 35256598 PMC8901805

[52] Seifu CN, Fahey PP, Hailemariam TG, Frost SA, Atlantis E. Dietary patterns associated with obesity outcomes in adults: an umbrella review of systematic reviews. Public Health Nutr. 2021;24(18):6390–414. doi: 10.1017/S1368980021000823 33612135 PMC11148623

[53] Tsompanaki E, Thanapirom K, Papatheodoridi M, Parikh P, Chotai de Lima Y, Tsochatzis EA. Systematic review and meta-analysis: the role of diet in the development of nonalcoholic fatty liver disease. Clin Gastroenterol Hepatol. 2023;21(6):1462-1474.e24. doi: 10.1016/j.cgh.2021.11.026 34838723

[54] Mega A, Marzi L, Kob M, Piccin A, Floreani A. Food and nutrition in the pathogenesis of liver damage. Nutrients. 2021;13(4):1326. doi: 10.3390/nu13041326 33923822 PMC8073814

[55] Polyzos SA, Kountouras J, Mantzoros CS. Obesity and nonalcoholic fatty liver disease: from pathophysiology to therapeutics. Metabolism. 2019;92:82–97. doi: 10.1016/j.metabol.2018.11.014 30502373

[56] Chaney A. Obesity and nonalcoholic fatty liver disease. Nurs Clin North Am. 2021;56(4):543–52. doi: 10.1016/j.cnur.2021.07.009 34749893

[57] Sookoian S, Rotman Y, Valenti L. Genetics of metabolic dysfunction-associated steatotic liver disease: the state of the art update. Clin Gastroenterol Hepatol. 2024;22(11):2177-2187.e3. doi: 10.1016/j.cgh.2024.05.052 39094912 PMC11512675

[58] Manikandan P, Nagini S. Cytochrome P450 structure, function and clinical significance: a review. Curr Drug Targets. 2018;19(1):38–54. doi: 10.2174/1389450118666170125144557 28124606

[59] Li S, Wang C, Zhang X, Su W. Cytochrome P450 omega-hydroxylase 4a14 attenuates cholestatic liver fibrosis. Front Physiol. 2021;12:688259. doi: 10.3389/fphys.2021.688259 34135776 PMC8201794

[60] Zhang X, Li S, Zhou Y, Su W, Ruan X, Wang B, et al. Ablation of cytochrome P450 omega-hydroxylase 4A14 gene attenuates hepatic steatosis and fibrosis. Proc Natl Acad Sci U S A. 2017;114(12):3181–5. doi: 10.1073/pnas.1700172114 28270609 PMC5373383

[61] Bergeron MJ, Clémençon B, Hediger MA, Markovich D. SLC13 family of Na⁺-coupled di- and tri-carboxylate/sulfate transporters. Mol Aspects Med. 2013;34(2–3):299–312. doi: 10.1016/j.mam.2012.12.001 23506872

[62] Martínez-Reyes I, Chandel NS. Mitochondrial TCA cycle metabolites control physiology and disease. Nat Commun. 2020;11(1):102. doi: 10.1038/s41467-019-13668-3 31900386 PMC6941980

[63] Birkenfeld AL, Lee H-Y, Guebre-Egziabher F, Alves TC, Jurczak MJ, Jornayvaz FR, et al. Deletion of the mammalian INDY homolog mimics aspects of dietary restriction and protects against adiposity and insulin resistance in mice. Cell Metab. 2011;14(2):184–95. doi: 10.1016/j.cmet.2011.06.009 21803289 PMC3163140

[64] von Loeffelholz C, Lieske S, Neuschäfer-Rube F, Willmes DM, Raschzok N, Sauer IM, et al. The human longevity gene homolog INDY and interleukin-6 interact in hepatic lipid metabolism. Hepatology. 2017;66(2):616–30. doi: 10.1002/hep.29089 28133767 PMC5519435

[65] Schumann T, König J, Henke C, Willmes DM, Bornstein SR, Jordan J, et al. Solute carrier transporters as potential targets for the treatment of metabolic disease. Pharmacol Rev. 2020;72(1):343–79. doi: 10.1124/pr.118.015735 31882442

[66] Pajor AM, Randolph KM. Inhibition of the Na+/dicarboxylate cotransporter by anthranilic acid derivatives. Mol Pharmacol. 2007;72(5):1330–6. doi: 10.1124/mol.107.035352 17715401

[67] Sun J, Aluvila S, Kotaria R, Mayor JA, Walters DE, Kaplan RS. Mitochondrial and plasma membrane citrate transporters: discovery of selective inhibitors and application to structure/function analysis. Mol Cell Pharmacol. 2010;2(3):101–10. 20686672 PMC2913483

[68] Ouldamer L, Jourdan M-L, Pinault M, Arbion F, Goupille C. Accumulation of arachidonic acid, precursor of pro-inflammatory eicosanoids, in adipose tissue of obese women: association with breast cancer aggressiveness indicators. Biomedicines. 2022;10(5):995. doi: 10.3390/biomedicines10050995 35625732 PMC9138452

[69] Berasain C, Avila MA. Vitamin A in nonalcoholic fatty liver disease: a key player in an offside position? Cell Mol Gastroenterol Hepatol. 2021;11(1):291–3. doi: 10.1016/j.jcmgh.2020.08.007 32971038 PMC7768560

[70] Markovich D. Physiological roles and regulation of mammalian sulfate transporters. Physiol Rev. 2001;81(4):1499–533. doi: 10.1152/physrev.2001.81.4.1499 11581495

[71] Chai X, Guo Y, Jiang M, Hu B, Li Z, Fan J, et al. Oestrogen sulfotransferase ablation sensitizes mice to sepsis. Nat Commun. 2015;6:7979. doi: 10.1038/ncomms8979 26259151 PMC4532951

[72] Kuroki T, Ikuta T, Kashiwagi M, Kawabe S, Ohba M, Huh N, et al. Cholesterol sulfate, an activator of protein kinase C mediating squamous cell differentiation: a review. Mutat Res. 2000;462(2–3):189–95. doi: 10.1016/s1383-5742(00)00036-3 10767630

[73] Puttaparthi K, Markovich D, Halaihel N, Wilson P, Zajicek HK, Wang H, et al. Metabolic acidosis regulates rat renal Na-Si cotransport activity. Am J Physiol. 1999;276(6):C1398-404. doi: 10.1152/ajpcell.1999.276.6.C1398 10362603

[74] Cissell DD, Link JM, Hu JC, Athanasiou KA. A modified hydroxyproline assay based on hydrochloric acid in Ehrlich’s solution accurately measures tissue collagen content. Tissue Eng Part C Methods. 2017;23(4):243–50. doi: 10.1089/ten.tec.2017.0018 28406755 PMC5397204

[75] Charni-Natan M, Goldstein I. Protocol for primary mouse hepatocyte isolation. STAR Protoc. 2020;1(2):100086. doi: 10.1016/j.xpro.2020.100086 33111119 PMC7580103

[76] Dobin A, Davis CA, Schlesinger F, Drenkow J, Zaleski C, Jha S, et al. STAR: ultrafast universal RNA-seq aligner. Bioinformatics. 2013;29(1):15–21. doi: 10.1093/bioinformatics/bts635 23104886 PMC3530905

[77] Love MI, Huber W, Anders S. Moderated estimation of fold change and dispersion for RNA-seq data with DESeq2. Genome Biol. 2014;15(12):550. doi: 10.1186/s13059-014-0550-8 25516281 PMC4302049

[78] Yu G, Wang L-G, Han Y, He Q-Y. clusterProfiler: an R package for comparing biological themes among gene clusters. OMICS. 2012;16(5):284–7. doi: 10.1089/omi.2011.0118 22455463 PMC3339379

