# Supplementary material for: Humanized monoacylglycerol acyltransferase 2 mice on a high-fat diet exhibit impaired liver detoxification during metabolic dysfunction-associated steatotic liver disease
Source: PLoS One. 2025 Oct 15;20(10):e0334213. doi: 10.1371/journal.pone.0334213 (PMC12527207; doi:10.1371/journal.pone.0334213)
Supplement: S1 Table — The source, catalog number, and dilution ratio, used for each antibody are listed. (DOCX) [file pone.0334213.s001.docx]

Table S1. Antibodies used for western analysis

| Protein | Company | Catalog# | Dilution |
| --- | --- | --- | --- |
| Srebp1 | Affinity BioSciences | BF8311 | 1:1:750 |
| Srebp2 | Abcam | ab30682 | 1:750 |
| pJak2 | Cell Signaling | 66245 | 1:1,000 |
| Jak2 | Cell Signaling | 74987 | 1:1,000 |
| pStat3 | Cell Signaling | 9145 | 1:1,000 |
| Stat3 | ThermoFisher | MA5-15712 | 1:2,500 |
| Cyp2b10 | Millipore Sigma | 9916 | 1:1,000 |
| Cyp3a11 | MYBioScience | MBS2132411 | 1:1,500 |
| Slc13a1 | ThermoFisher | PA5-62818 | 1:1,000 |
| Slc13a2 | ThermoFisher | PA5-76396 | 1:1,000 |
| Gapdh | Millipore Sigma | 2118 | 1:1,000 |
| Lamin | Cell Signaling | 4777 | 1:2,000 |
|  |  |  |  |
|  |  |  |  |
|  |  |  |  |
|  |  |  |  |
|  |  |  |  |
|  |  |  |  |
|  |  |  |  |
|  |  |  |  |
|  |  |  |  |
|  |  |  |  |
