# Supplementary material for: Humanized monoacylglycerol acyltransferase 2 mice on a high-fat diet exhibit impaired liver detoxification during metabolic dysfunction-associated steatotic liver disease
Source: PLoS One. 2025 Oct 15;20(10):e0334213. doi: 10.1371/journal.pone.0334213 (PMC12527207; doi:10.1371/journal.pone.0334213)
Supplement: S2 Table — The source and catalog number for each ELISA kit are listed. (DOCX) [file pone.0334213.s002.docx]

Table S2. Commercial ELISA Kits used in this study

| Protein | Company | Catalog# |
| --- | --- | --- |
| pAkt | Abcam | ab126433 |
| Akt | Abcam | ab126433 |
| pS6 | Cell Signaling | 13911 |
| S6 | LSBio | LS-F1616 |
| pGsk3β | Abcam | ab279814 |
| Gsk3β | RayBiotech | ELM-Gsk3b-1 |
| Col1a1 | Abcam | ab210579 |
| Col3a1 | LS-BIO | LS-F51703-1 |
| Mmp2 | Abcam | ab254516 |
| Mmp9 | Abcam | ab253227 |
| Mmp12 | Abcam | ab246540 |
| Mmp13 | R&D Systems | DY918-05 |
| Galectin-3 | Abcam | ab203369 |
| mouse pJak2 | Cell Signaling | 63125 |
| mouse Jak2 | Abcam | Ab25322 |
| human pJak2 | RayBiotech | PEL-JAK2-Y1007-T-1 |
| human Jak2 | ThermoFisher | KH05521 |
| mouse pStat3 | Abcam | ab126459 |
| mouse Stat3 | Abcam | ab126459 |
| human pStat3 | Abcam | ab126459 |
| human Stat3 | Abcam | ab126459 |
| Bax | Abcam | ab233624 |
