# Supplementary figures and images for: Humanized monoacylglycerol acyltransferase 2 mice on a high-fat diet exhibit impaired liver detoxification during metabolic dysfunction-associated steatotic liver disease
Source: PLoS One. 2025 Oct 15;20(10):e0334213. doi: 10.1371/journal.pone.0334213 (PMC12527207; doi:10.1371/journal.pone.0334213)

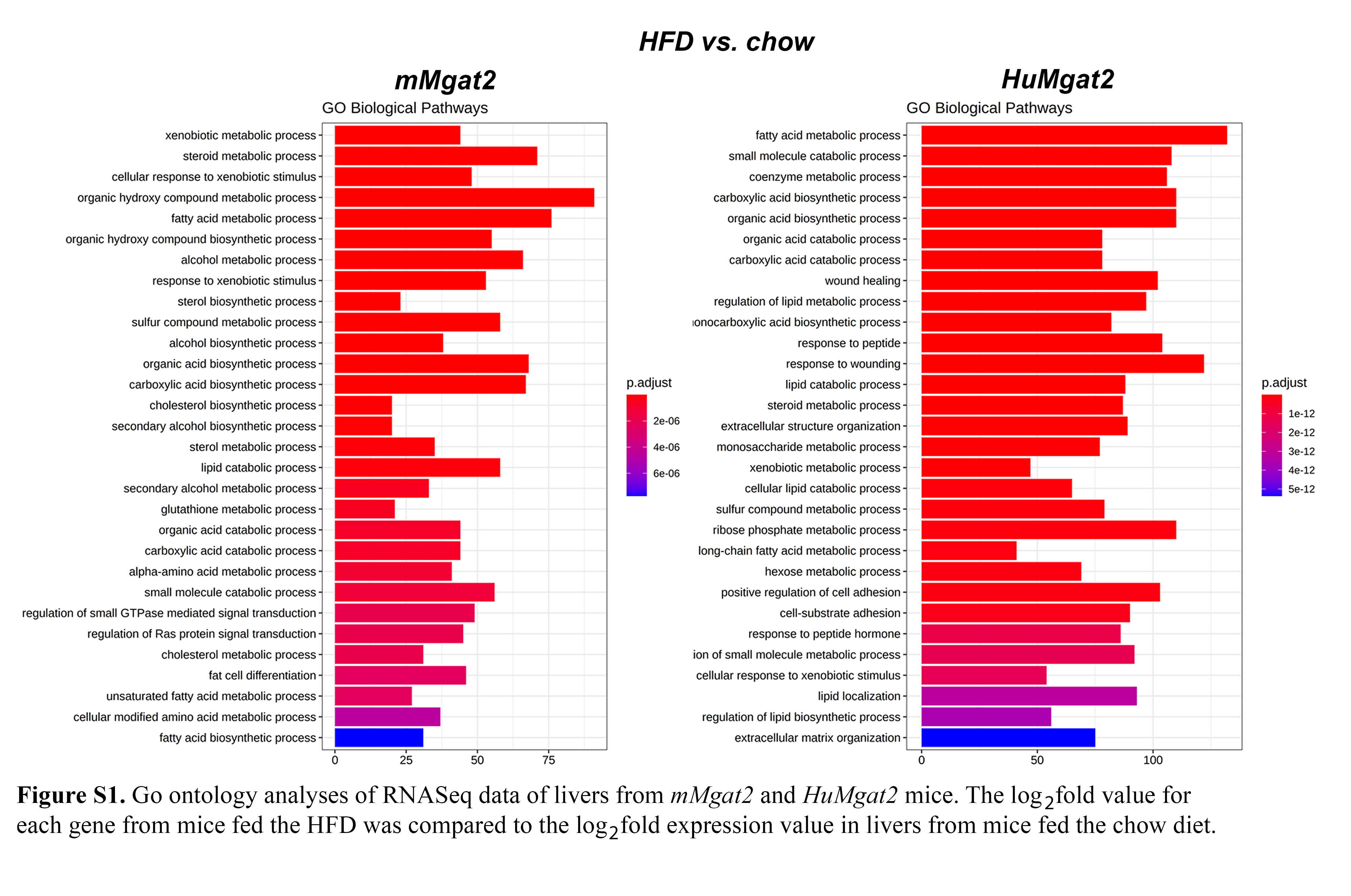

Supplement: S1 Fig — The log2fold value for each gene from mice fed the HFD was compared to the log2fold expression value in livers from mice fed the chow diet. (TIF) [file pone.0334213.s003.tif]

*mMgat2*

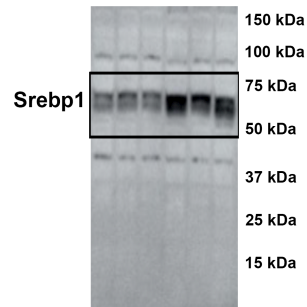

*HuMgat2*

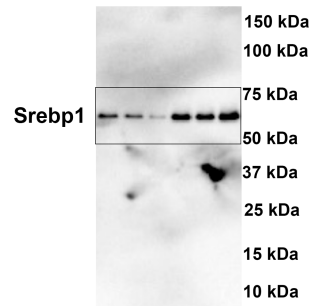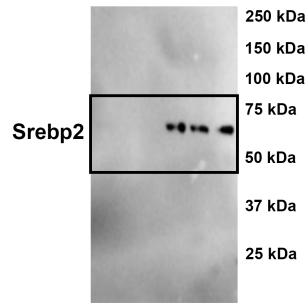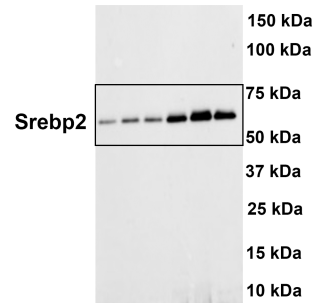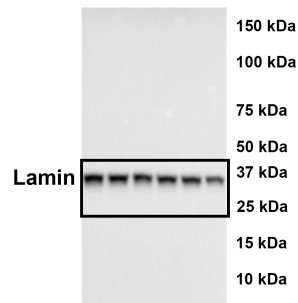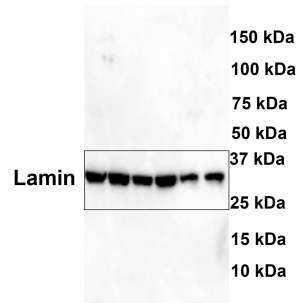

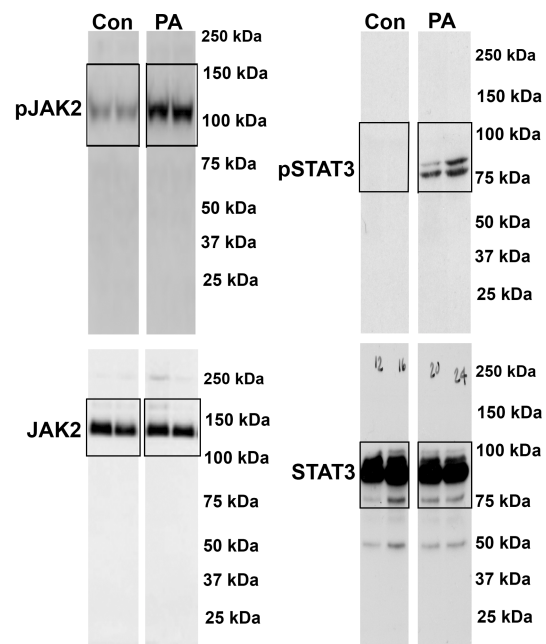

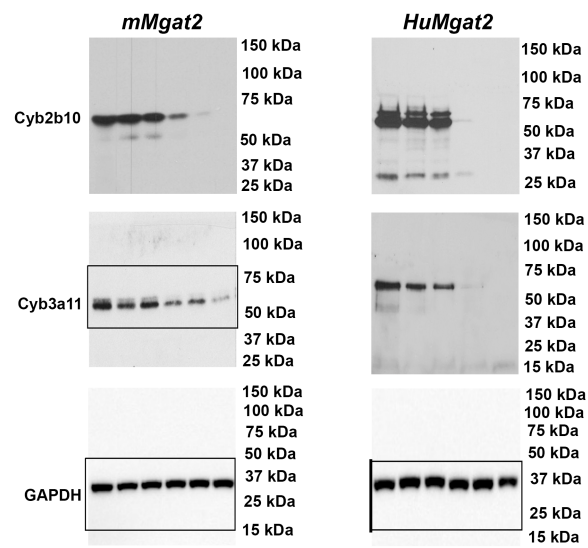

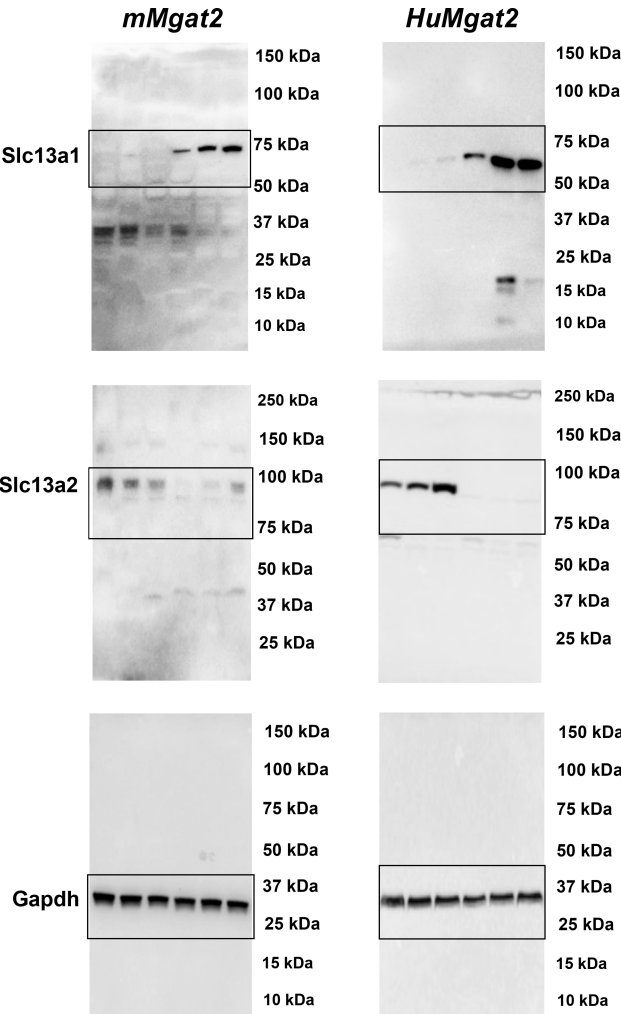

Supplement: S2 Fig — Full-length western blots used to construct western figure panels. (PDF) [file pone.0334213.s004.pdf]

*mMgat2*

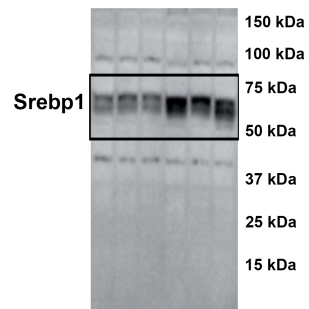

*HuMgat2*

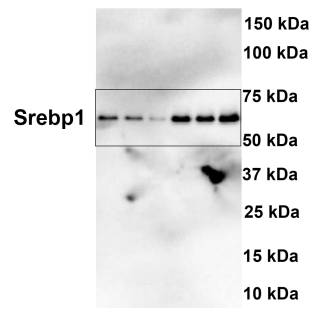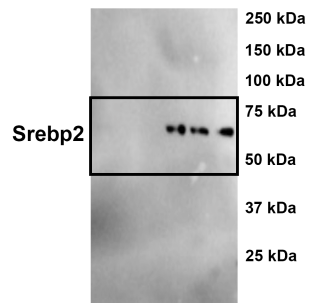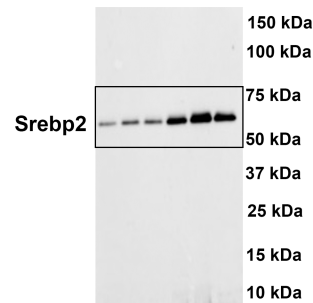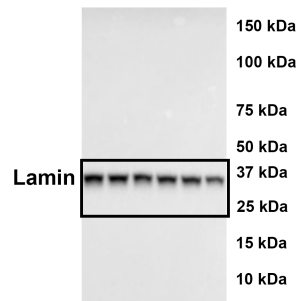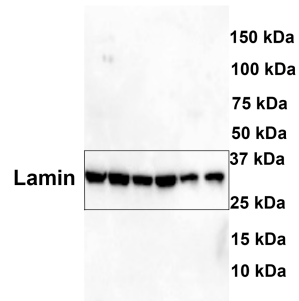

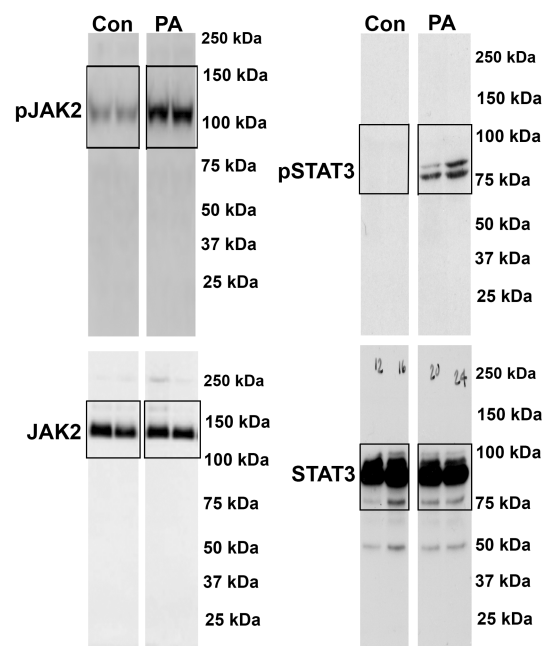

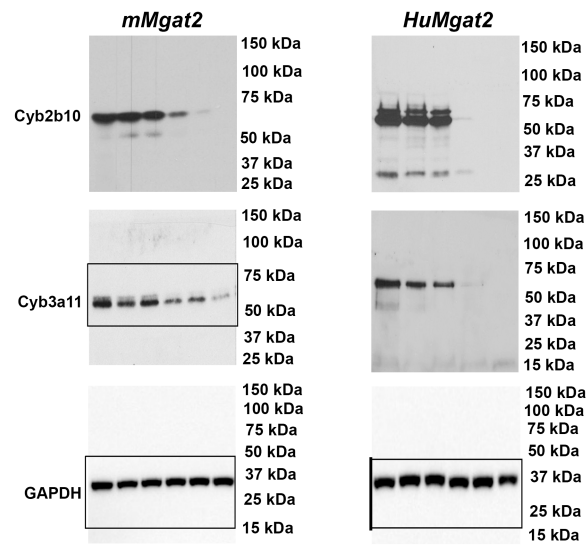

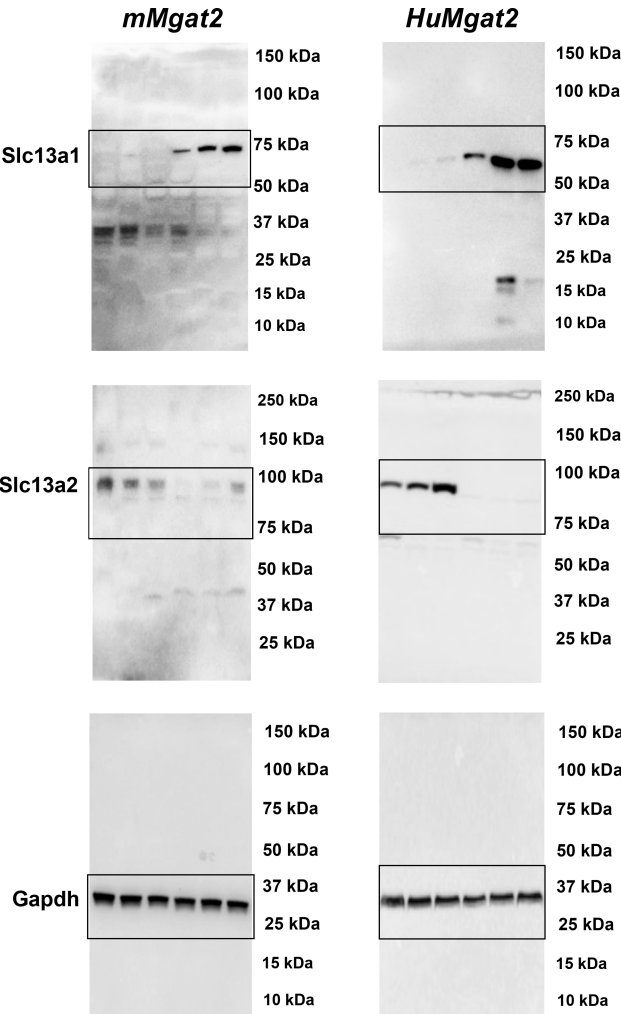

Supplement: S1 File — (PDF) [file pone.0334213.s005.pdf]
